# Evolutionary Patterns of Bone Histology and Bone Compactness in Xenarthran Mammal Long Bones

**DOI:** 10.1371/journal.pone.0069275

**Published:** 2013-07-09

**Authors:** Fiona R. Straehl, Torsten M. Scheyer, Analía M. Forasiepi, Ross D. MacPhee, Marcelo R. Sánchez-Villagra

**Affiliations:** 1 Paläontologisches Institut und Museum, Universität Zürich, Zürich, Switzerland; 2 CONICET, IANIGLA, CCT-Mendoza, Mendoza, Argentina; 3 Vertebrate Zoology, American Museum of Natural History, New York, New York, United States of America; Raymond M. Alf Museum of Paleontology, United States of America

## Abstract

Bone microstructure reflects physiological characteristics and has been shown to contain phylogenetic and ecological signals. Although mammalian long bone histology is receiving increasing attention, systematic examination of the main clades has not yet been performed. Here we describe the long bone microstructure of Xenarthra based on thin sections representing twenty-two species. Additionally, patterns in bone compactness of humeri and femora are investigated. The primary bone tissue of xenarthran long bones is composed of a mixture of woven, parallel-fibered and lamellar bone. The vascular canals have a longitudinal, reticular or radial orientation and are mostly arranged in an irregular manner. Concentric rows of vascular canals and laminar organization of the tissue are only found in anteater bones. The long bones of adult specimens are marked by dense Haversian bone, a feature that has been noted for most groups of mammals. In the long bones of armadillos, secondary osteons have an oblique orientation within the three-dimensional bone tissue, thus resulting in their irregular shape when the bones are sectioned transversely. Secondary remodeling is generally more extensive in large taxa than in small taxa, and this could be caused by increased loading. Lines of arrested growth are assumed to be present in all specimens, but they are restricted to the outermost layer in bones of armadillos and are often masked by secondary remodeling in large taxa. Parameters of bone compactness show a pattern in the femur that separates Cingulata and Pilosa (Folivora and Vermilingua), with cingulates having a lower compactness than pilosans. In addition, cingulates show an allometric relationship between humeral and femoral bone compactness.

## Introduction

The bone microstructure of the vertebrate skeleton has been studied since the nineteenth century, with Quekett [1], [2], among others, laying the foundation for further research into comparative osteohistology. Later research included both fossil and extant samples, and provided a better overview of microscopic structures of bone among major groups of vertebrates (e.g. [3]–[6]). Cubo et al. [7] demonstrated that bone microstructure, like many other biological features, is the product of historical (phylogenetic), functional and structural factors. Bone microstructure does, in addition, also vary greatly over the course of the developmental history of an organism. Among functional factors, parameters of locomotion [8], metabolism [9], and lifestyle [10]–[14] have been analyzed. Lifestyle is mainly investigated via quantitative analyses of bone tissue by looking at properties such as cross-sectional geometry [15] or bone compactness [16]. Bone compactness parameters have been analyzed in a variety of vertebrates [10]–[13], [17]–[19], and have shown to contain phylogenetic and ecological signals. These studies were, however, all conducted at a broad scale. Recently, Meier et al. [20] investigated bone compactness within a narrower clade, the moles (Talpidae), and found no significant differences between fossorial and non-fossorial taxa.

Bone histology of mammals in general has been investigated in the past, but in most cases only as a portion of a larger scale study of vertebrates (e.g. [2], [5], [21]). Studies focusing solely on the histology of extinct and extant mammals are still relatively rare, but important works published so far include those of Singh et al. [22], Klevezal [23], Sander and Andrássy [24] and Köhler et al. [25]. One of the most significant findings of these studies was the presence of lines of arrested growth (LAGs), a feature that was for a long time believed to be exclusive to ectotherms, the new reports thus raising questions about the ectothermic physiology that had once been hypothesized for dinosaurs [24]–[27]. But despite the increasing interest in mammalian bone microstructure and the promising results gained so far, little effort has been made to systematically investigate and compare mammalian microscopic bone features.

The Xenarthra represent one of the four major clades of placental mammals [28], and comprise extant armadillos, sloths and anteaters, and their extinct relatives. At present, 31 species of living xenarthrans are recognized, comprising six sloths, four anteaters, and 21 species of armadillos [29]. Most extant xenarthrans are found in Latin America, with the majority being endemic to South America. *Dasypus novemcinctus*, the nine-banded armadillo, has invaded and successfully colonized much of southern North America within the last two hundred years [30]. Xenarthra most likely evolved in South America, and the diversity of fossil taxa is very high, with more than 150 genera known [31] and with great morphological diversity.

**Figure 1:**
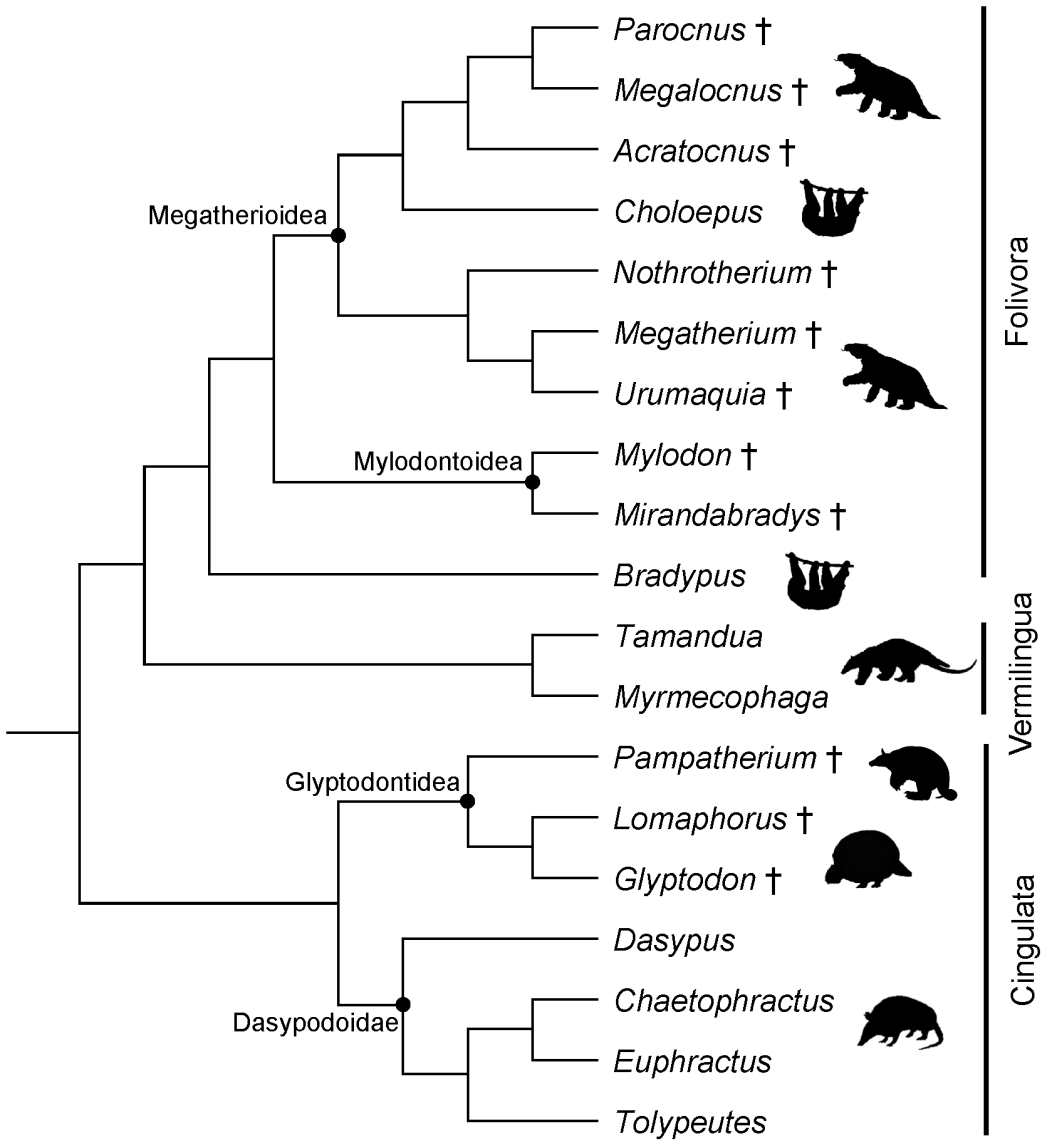
Phylogenetic relationships between taxa included in this study. The phylogeny is based on [96] and [38], and placement of some fossil taxa follows [31].

Phylogenetic reconstructions estimated the origin of Xenarthra to be before or close to the Cretaceous-Paleogene boundary, about 80–65 mya [32]–[36]. On the basis of a combined phenomic-molecular analysis, O'Leary et al. [36] recently found high support for a split of Xenarthra from all other placental mammals (Epitheria) shortly after the Cretaceous-Paleogene boundary. Since then, three ecologically disparate and phylogenetically well supported groups have evolved within Xenarthra [37] (Figure 1). Cingulata includes modern armadillos and the extinct glyptodonts and pampatheres. Members of this group possess an armor that covers head, body and tail. In glyptodonts this armor is rigid, while it has movable bands in armadillos and pampatheres. Despite this morphological similarity to armadillos, the pampatheres are hypothesized to be more closely related to glyptodonts (e.g. [38], [39]). The remaining two groups of xenarthrans, Vermilingua (anteaters) and Folivora (sloths), both lack armor and are widely considered to group together as Pilosa, based on morphological and molecular data [32], [37], [38], [40]–[43]. The Vermilingua feature elongate, tubular skulls, an absence of teeth, and a prehensile tongue used for the acquisition of prey. The Folivora are characterized by a hypselodont herbivorous dentition and a reduced dental formula that is exclusive to this group [43]. Both ground and tree sloths lack incisors and true canines, and their cheek teeth are restricted to the maxilla [44]. Folivorans are further well known for their very low metabolism, a feature that is actually common to all xenarthrans, and for their slow locomotion. These features have traditionally also been assumed for their fossil relatives [45]. More recent discoveries and analyses of their functional morphology have suggested more diverse lifestyles for some extinct folivorans, including aquatic habits [46].

Xenarthrans show a marked pattern of disparity in many biological parameters. Differences in body size between extinct and extant taxa are striking, with the weight of glyptodonts estimated at one to two tons and the weight of the giant ground sloth *Megatherium americanum* at almost six tons [47]. Recent studies have detected an unusually high degree of morphological variation within Xenarthra. Asher et al. [48] found substantial variation in vertebral counts, and Rager and Sánchez-Villagra [49] noted high variability in the timing of cranial suture closure. The latter is especially high among extant sloths, for which notable intraspecific variation has also been found in body size [50] and in the inner ear morphology [51] of species of *Bradypus.* On the microscopic level, the histology of osteoderms of cingulates and the mylodontids showed interspecific variation [52]. Since dermal ossifications provide information on soft tissue structures otherwise not preserved in fossils, such studies on the histology of xenarthran osteoderms have also shed light on phylogenetic relationships within the group and on the functional morphology of xenarthrans [39], [52]–[57]. Within Pilosa, only species of mylodontid sloths are known to have possessed osteoderms (e.g. [58], [59]).

This study represents the first fine-scale investigation into the bone histology and bone compactness of Xenarthra, with long bones representing all three major clades analyzed qualitatively and quantitatively. Our aim is to address the following questions: Do the histology and/or the compactness of xenarthran long bones show any patterns that could be related to the variation in body size or locomotory modes found in this clade? Do the histology and/or compactness of long bones of Cingulata and Pilosa differ substantially, e.g. due to the occurrence of armour in the former group? Does the long bone histology of xenarthrans comply with the general pattern of disparity for which the group is known?

## Materials and Methods

### Ethics Statement

All bones sampled for this study were loaned from the below-mentioned institutions after getting permission to access the collections.

### Institutional Abbreviations

AMNH, American Museum of Natural History, New York, USA; AMU-CURS, Colección de Paleontología de Vertebrados de la Alcaldía de Urumaco, Estado Falcón, Venezuela; MHNSR-PV, Museo de Historia Natural de San Rafael (Paleontología de Vertebrados), San Rafael, Mendoza, Argentina; NMB, Naturhistorisches Museum Basel, Basel, Switzerland; PIMUZ, Paläontologisches Institut und Museum der Universität Zürich, Zürich, Switzerland; ZMZ, Zoologisches Museum der Universität Zürich, Zürich, Switzerland.

### Material Sampled

A total of sixty-seven mostly adult long bones (humerus, femur, radius, ulna, and tibia) of fossil and extant xenarthrans, representing nineteen genera and twenty-two species, were sampled (Table 1, Table S1). Fossil samples represent only a small subset of the large diversity of extinct xenarthrans [60]–[65], and they illustrate the spectrum of extinct diversity. Long bones are the most abundant remains of fossil xenarthrans, and they are well suited for future comparative work since the emphasis of studies on mammalian bone histology and tetrapod bone microanatomy has traditionally been on these bones (e.g. [10]–[13], [17], [19], [22], [24]).

**Table 1:** List of taxa studied, including taxonomic information and catalogue numbers.

Taxonomy	Taxon	Reference No.	Elements sampled
Glyptodontidae	*† Glyptodon clavipes*	PIMUZ A/V 463	humerus, radius, ulna
		PIMUZ A/V 465	humerus, radius, ulna
	*† Lomaphorus ornatus*	PIMUZ A/V 438	humerus, radius, ulna, femur
Pampatheriidae	*† Pampatherium typum*	PIMUZ A/V 428	humerus, radius
Dasypodidae	*Chaetophractus vellerosus*	ZMZ 20213	humerus, radius, ulna, femur
	*Dasypus hybridus*	PIMUZ A/V 4798	humerus, femur
		PIMUZ A/V 4799	humerus, femur
	*Dasypus novemcinctus*	PIMUZ A/V 4800	humerus, femur
		PIMUZ A/V 4801	humerus, femur
		PIMUZ A/V 4802	humerus
	*Euphractus sexcinctus*	ZMZ 17834	humerus, femur
		PIMUZ A/V 4803	humerus, femur
		PIMUZ A/V 4804	humerus, femur
	*Tolypeutes matacus*	ZMZ 11151	humerus, femur
	*Tolypeutes tricinctus*	PIMUZ A/V 4805	humerus, femur
		PIMUZ A/V 4806	humerus, femur
Megalonychidae	*† Acratocnus antillensis*	AMNH u1	tibia
	*† Megalocnus rodens*	AMNH u2	femur
	*† Parocnus brownii*	AMNH u3	humerus
	*Choloepus didactylus*	ZMZ 17223	humerus, radius, ulna, femur
Megatheriidae	*† Megatherium* sp.	AMU-CURS 220*	femur
	*† Urumaquia robusta*	AMU-CURS 169*	femur
Mylodontidae	*† Mirandabradys zabasi*	AMU-CURS 128	femur
	*† Mylodon robustus*	PIMUZ A/V 501	tibia
Nothrotheriidae	*† Nothrotherium escrivanse*	PIMUZ A/V 477	humerus, radius, ulna, tibia
	† Folivora indet.	MHNSR-PV 1002*	femur
		MHNSR-PV 1003*	humerus
		MHNSR-PV 1006*	radius
		MHNSR-PV 1007*	radius
Bradypodidae	*Bradypus torquatus*	ZMZ 11102	humerus, femur
	*Bradypus tridactylus*	NMB 10488	humerus, femur
Myrmecophagidae	*Tamandua tetradactyla*	NMB 10420	humerus, femur
		PIMUZ A/V 4807	humerus, femur
		PIMUZ A/V 4808	humerus, femur
	*Myrmecophaga tridactyla*	ZMZ 11119	humerus, femur

* Core samples.

### Methods of Sectioning

**Figure 2:**
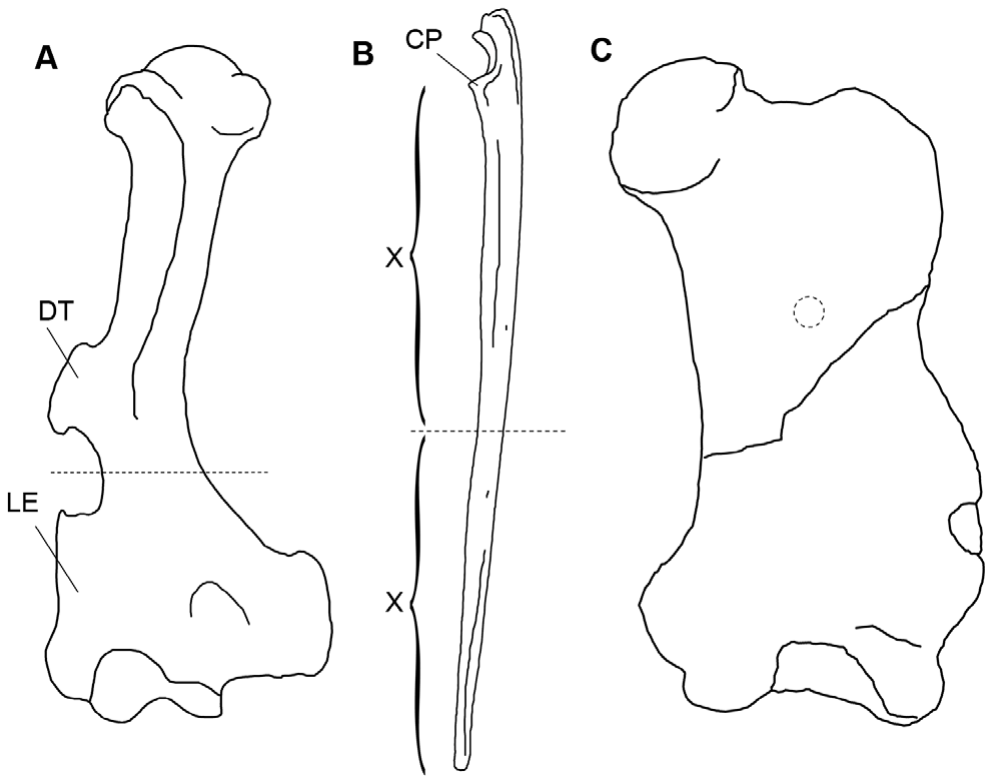
Schematic drawings of xenarthran long bones showing alternative sampling sites (dashed lines). **A.** Right humerus of PIMUZ A/V 4807 *Tamandua tetradactyla,* sectioned between the deltoid tubercle (DT) and the lateral epicondyle (LE). **B.** Right ulna of ZMZ 17223 *Choloepus didactylus,* sectioned at halfway distance from the coronoid process (CP) to the most distal point of the bone. **C.** Left femur of AMU-CURS 169 *Urumaquia robusta,* sectioned by core-drilling in the proximal half of the bone so as to avoid the mediolateral crack. Note that bones are not drawn to scale.

Whenever possible, bones were sampled at the mid-diaphyseal level. Long bones undergo the least secondary remodeling at this level, yielding the most complete growth record [66], [67]. Additionally, the mid-diaphyseal level has shown the maximum ecological signal [17]. With regard to the bone compactness analysis, this also ensured that variation in compactness profiles of different bones was not due to variation in connection with different sampling sites. In most cases, the mid-diaphyseal level corresponded to the area where the bone exhibited the smallest circumference. However, the sampling site had to be adjusted for humeri of anteaters and for all xenarthran ulnae (Figure 2). Anteater humeri are characterized by a prominent deltoid tubercle located in the mid-diaphyseal region. Because bone drift and bone remodeling typically occur in such regions of bony outgrowth, sectioning through the deltoid tubercle would produce thin sections showing a distorted growth record of the humerus. Anteater humeri were thus sampled in the area with the least circumference between the deltoid tubercle and the lateral epicondyle. The sampling sites also had to be adjusted for ulnae, because in xenarthrans these bones vary greatly in shape and relative size, and the area with the smallest circumference did not always correspond to the mid-diaphyseal level. Therefore, to standardize sampling sites in ulnae, we took the distance between the coronoid process and the distalmost end of the bone, and then cut the bone halfway through this distance. Fossil bones considered too valuable or too large for producing regular cross sections were sampled by core drilling (see also [68]). For this purpose a 20 mm diamond-studded core drill bit (Metabo SBE 610) at 300 RPM was used.

For the production of thin sections, we followed standard procedures (e.g. [69], [70]). Prior to sectioning, high-resolution photographs were taken, using a Nikon® D2× digital reflex camera. Bones from the collection of the Zoologisches Museum Universität Zürich were additionally molded and cast. The molding agents used during this project were Elastosil® M 4503 and Wacker® Catalyst T 46 (Ameba AG, CH-4056 Basel). The sampling sites of all extant and some fossil bones were mantled with either a two-component resin (Technovit® 5071), superglue, or, in the case of some MNHSR-PV samples, paraffin wax to prevent additional damage during sawing and drilling. Core samples were embedded in transparent two-component epoxy resin (Araldite® 2020 XW 396/397, Huntsman A.M., B-Everberg). To produce thin sections, we cut the bones and the embedded core samples in half, using a rock saw (Diamant Boart) for fossil bones, a diamond blade saw for core samples (Hartig Export), and a band-saw (Inland DB-100™) for bones of extant taxa. After letting the samples dry for at least 24 hours, cut surfaces were impregnated with transparent two-component epoxy resin (Araldite® 2020 XW 396, Huntsman A.M., B-Everberg), under vacuum conditions when possible. Most bones, however, were impregnated by applying a sufficient amount of resin to the cut surface and then placing the bone with the cut surface facing down on a piece of sheet protector attached to the tabletop. Once the resin had been allowed to harden completely, we polished the impregnated surfaces manually using a vertical or horizontal grinding disc (Crystal Master 8) and silicon carbide powder of variable grain sizes (220, 500, 800 grit, IEPCO AG, CH-Höri). One half of each core sample was additionally polished into a polished section sensu Sander [69] using 1200 grit silicon carbide powder and cerium(IV) oxide powder applied on leather. Polished samples intended for thin sections were then attached to roughened glass slides, using Araldite®. As a next step, we cut sections of 1 to 2 mm thickness using a diamond blade saw. These sections were reduced to a thickness of 100 to 150 µm with a sintered diamond grinding wheel (Wendt LM3). Then the sections were polished by hand to a final thickness of 60 to 80 µm. A cover slip was applied using UV glue (Vitralit® 6127, Panacol AG, CH-Regensdorf). Finally, thin sections were examined by standard light microscopy (normal and cross-polarized) and polished sections under bright field illumination using a composite microscope (Leica® DM2500 M). Photographs of histological features were taken with a Leica® DFC 420 C digital camera mounted on the Leica® DM2500 M composite microscope. Thin sections of all sampled bones have been returned to the respective repositories together with the long bones, with the exception of the sections of AMNH u1, AMNH u2 and AMNH u3.

### Bone Compactness Profile Data

**Figure 3:**
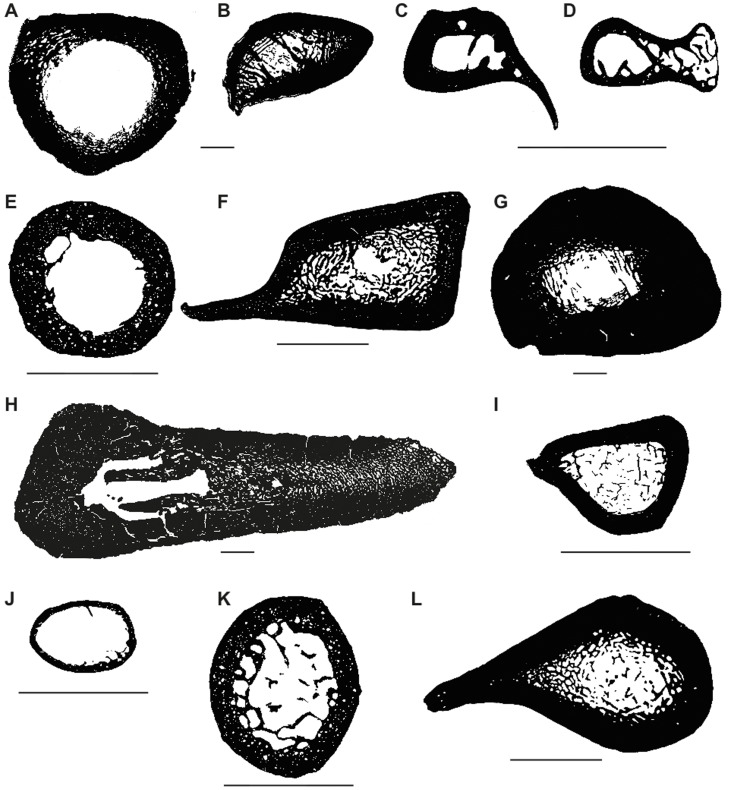
Selection of binary images produced for the compactness analysis showing the diversity of size and shape. **A–F.** Humeri. **G–L.** Femora. **A.** *Glyptodon clavipes* PIMUZ A/V 463. **B.** *Lomaphorus ornatus* PIMUZ A/V 438. **C.** *Dasypus novemcinctus* PIMUZ A/V 4802. **D.** *Tolypeutes matacus* ZMZ 11151. **E.** *Choloepus didactylus* ZMZ 17223. **F.** *Myrmecophaga tridactyla* ZMZ 11119. **G.** *Lomaphorus ornatus* PIMUZ A/V 438. **H.** *Mirandabradys zabasi* AMU-CURS 128. **I.** *Dasypus novemcinctus* PIMUZ A/V 4800. **J.** *Tolypeutes tricinctus* PIMUZ A/V 4806. **K.** *Choloepus didactylus* ZMZ 17223. **L.** *Myrmecophaga tridactyla* ZMZ 11119. Scale bar: 1 cm.

Bone compactness analysis was carried out for humeri and femora only, as these were the most abundant bones in the sample, especially for fossil taxa. Cross-sections were digitized using a flatbed scanner for big thin sections and a Leica® DFC 420 C digital camera mounted on a Leica® DM2500 M composite microscope for smaller thin sections. In most cases, the smaller thin sections had to be photographed partially, and images were then merged automatically in Adobe Photoshop® CS5 (Adobe Systems Inc., San Jose, CA, USA). We transformed the photographs into binary images in Adobe Photoshop® CS5 by marking bone in black and vascular spaces (medullary cavity, vascular canals, resorption spaces) in white. The binary images (Figure 3, Figures S1.1–S1.5) were analyzed quantitatively with the software Bone Profiler Version 4.5.6 [16], which can extract several parameters of a bone compactness profile from cross-sections. In brief, the program uses the bone center as the pivot point and estimates compactness in 60 radial sectors of 6° width, which are further divided into 51 concentric zones each. Compactness profiles were measured using the program’s automatic mechanism for the detection of the bone center. However, in some cases it was necessary to adjust ‘limit medulla’ and ‘section center’ manually. Bone Profiler then plots the data using bone compactness (*C*) as a function of the distance from the bone center (*d*):
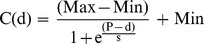



From this function, four main parameters are extracted by the program: the relative distance from the section center to the point of most abrupt change in compactness, i.e., transition from medulla to cortex (*P*); the reciprocal of the slope at the inflexion point (*S*); the upper and the lower asymptote of the function (*Max* and *Min* respectively). The latter two correspond to the compactness in the outermost cortex (*Max*) and the compactness in the center of the medullary cavity (*Min*). Bone Profiler additionally extracts observed compactness (or global compactness, *Cg*), among other parameters.

Global compactness (*Cg*) is the parameter with the most relevance to this study, which aims at detecting general patterns in bone compactness. However, Canoville and Laurin [13] detected the most significant ecological signal in parameters *S*, *P*, *Cc* (compactness in the bone center), and *Cp* (compactness in the periphery of the section) in a study of amniote long bones. Additional plots were therefore produced for parameters *S* and *P*, but not for *Cc* and *Cp*, because all thin sections analyzed in this study had *Cc* values around 0 and *Cp* values around 1. *S* also showed an ecological signal in a fine-scale study of bone compactness in talpid humeri, in that values were significantly larger for fossorial than for non-fossorial forms [20].

## Results

### Histological Features

The microstructure of long bones is described for two body mass categories of Cingulata, for two body mass categories of Folivora, and one category for Vermilingua. All descriptions follow the terminology of Francillon-Vieillot et al. [67]. In agreement with Werning [71], we refrain from using tissue-level terms like ‘fibro-lamellar bone’. Instead, we concentrate on the description of collagen orientation, vascular orientation, spatial arrangement of vascular canals, and osteocyte shape and arrangement. Body mass categories are based on data from Smith et al. [70] and White [71]. A summary of histological features found in xenarthran long bones is provided in Table 2. In addition to the histological features described below, Sharpey's fibers indicating various muscle attachments are found in the majority of the bones. We do, however, refrain from describing and discussing them any further, as this would go beyond the scope of this paper.

**Table 2:** Summary of vascularization and tissue types found in xenarthran long bones of different major clades and body mass categories.

Histological features		Cingulata		Folivora		Vermilingua
		>20 kg	<20 kg	>20 kg	<20 kg	5–22 kg
***Vascularization***	**orientation**	long, ret, rad	long, ret, rad	long, ret, elngtd	long, rad	long, ret, circ
	**arrangement**	irreg	irreg, circ	irreg	irreg	circ rows, irreg
***Tissue types***	**external layer**	pf and la, av	pf and la, almost av	pf and la, av	la, av	–
	**majority of cortex**	dhb	dhb, pf	dhb, pf and la/wpl	dhb, pf and la	dhb, wpl
	**innermost layer**	la, av	pf & la, av	la, av	la, av	la
	**trabeculae**	pf and la	pf and la	pf and la	la	pf and la

Body mass categories are based on data by Smith et al. [97] and White [98]. Abbreviations: av avascular; circ circular; dhb dense Haversian bone; elngtd elongated; irreg irregular; la lamellar; long longitudinal; pf parallel-fibered; rad radial; ret reticular; wpl woven, parallel and lamellar fiber arrangement.

#### Cingulata: species >20 kg (all extinct)

**Figure 4:**
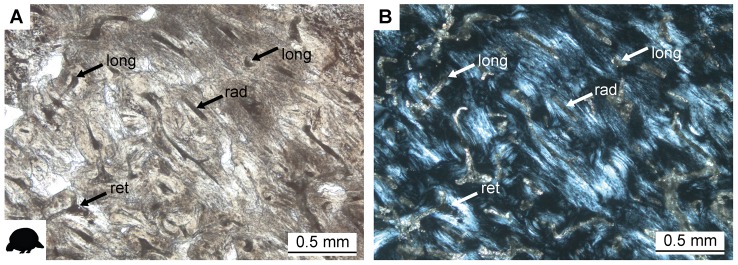
Bone histology of *Lomaphorus ornatus* PIMUZ A/V 438 (A–C) and *Glyptodon clavipes* PIMUZ A/V 463 (D–F). **A.** Bone tissue of femur, conforming to random arrangement of longitudinal (long), reticular (ret) and radial (rad) vascular canals. Normal light. **B.** The same as in A in cross-polarized light.

Long bones of large cingulates are characterized by a compact cortex and a dense network of thick trabeculae in the medullary region. In some samples, trabeculae had been previously destroyed by diagenetic processes, or were damaged during bone sectioning. The humerus of *Pampatherium typum* PIMUZ A/V 428 exhibits a high spatial organization of cancellous bone anteriorly, where three thin and long trabeculae are arranged parallel to each other. The cortices of the ulnae are thin in comparison to the cortices of other long bones included in this sample. Orientation of the vascular canals in the cortical tissue of cingulate long bones varies among the different skeletal elements. In the humeri, orientation of the vascular canals is variably longitudinal, radial, or reticular. In all other elements, vascular canals are mainly oriented longitudinally, with reticular canals occasionally occurring in the femur. The arrangement of vascular canals is, however, irregular in all elements. The cortices of the radii of *Glyptodon clavipes*, PIMUZ A/V 463 and PIMUZ A/V 465, show expansion of the cancellous medullary bone into the posterior region of the cortex. Most of the cortex has been remodeled and dense Haversian bone is visible under cross-polarized light. Because the vascular canals are variously orientated in some areas of the bones, the remodeled tissue locally appears irregular (Figure 4a–b). Due to the preservational state, osteocyte lacunae are hardly visible. A thin layer of parallel-fibered and, in certain areas, lamellar bone tissue surrounds the exterior cortex. The medulla is lined by a thin strip of primary lamellar bone, and trabeculae show parallel-fibered and lamellar tissue organization.

#### Cingulata: species <20 kg (all extant)

**Figure 5:**
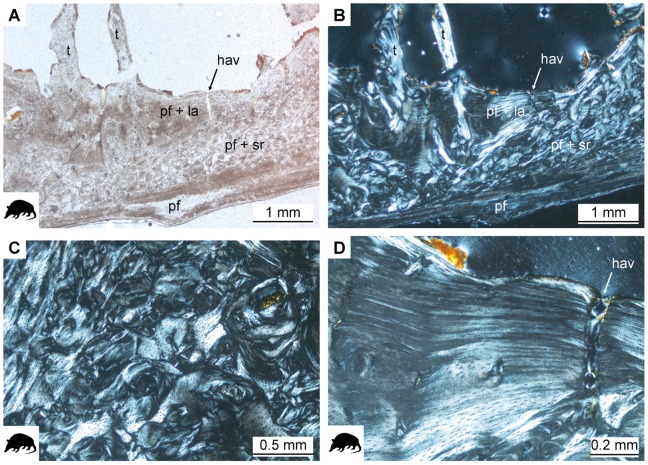
Histology of the humerus of *Euphractus sexcinctus* ZMZ 17834. **A.** Overview of the three layers with different tissue types present in the cortex under normal light. **B.** The same as in A in cross-polarized light. **C.** Close-up on the dense Haversian tissue with irregularly shaped secondary osteons under cross-polarized light. **D.** Close-up on the internal layer of parallel-fibered and lamellar tissue under cross-polarized light. hav radial Haversian system, la lamellar tissue, pf parallel-fibered tissue, sr secondary remodeling.

The long bones of all sampled armadillos possess a thick, compact cortex. Only *Dasypus hybridus* PIMUZ A/V 4799 and *Tolypeutes tricinctus* PIMUZ A/V 4806 show thin cortices, and these two specimens were identified as juveniles on the basis of the level of epiphyseal fusion. The cortex shows three major layers of different bone tissues (Figure 5a–b). A layer of poorly vascularized parallel-fibered bone tissue lines the cortex at the periphery. This layer is of variable thickness within and between bones and sparsely vascularized. In most cases, a very thin layer of lamellar bone has been deposited external to this layer of parallel-fibered bone. This outer layer (OCL: outer circumferential layer or EFS: external fundamental system as discussed by Chinsamy [72]) is clearly delimited by a line representing a LAG or annulus. Within the OCL up to five closely spaced LAGs (or annuli) were found in adult specimens of armadillos (all except PIMUZ A/V 4799 and PIMUZ A/V 4806).

Arrangement of vascular canals in the parallel-fibered middle layer is random, and canals are partly oriented longitudinally, radially and reticularly. Only in certain areas of the humeri of *Dasypus novemcinctus* (PIMUZ A/V 4800, PIMUZ A/V 4801, PIMUZ A/V 4802) and *Euphractus sexcinctus* ZMZ 17834 are vascular canals arranged in circular rows. Remodeling varies in its extent between the sampled individuals of armadillos and is most pronounced in ZMZ 17834, where dense Haversian bone is found. Secondary osteons are poorly differentiated and in certain areas irregularly shaped (Figure 5c). The convoluted shape of secondary osteons visible in Figure 5c could thus result from their oblique orientation within the three-dimensional bone tissue architecture. The cancellous medulla is lined by an avascular layer of secondarily deposited parallel-fibered and lamellar tissue, varying very much in thickness within a single bone (Figure 5d). A loose network of thick trabeculae, often extending across the entire medullary region, forms the cancellous region of the humeri. These trabeculae are composed of parallel-fibered and lamellar bone tissue. In the radius and ulna of *Chaetophractus vellerosus* ZMZ 20213 trabeculae are absent, and in femora trabeculae are thin and form a dense network. Both sections of the humerus of *Tolypeutes matacus* ZMZ 11151 and the more proximal section of the humerus of *Dasypus hybridus* PIMUZ A/V 4799 show that the cancellous region extends into the cortex in the region of the deltoid tuberosity.

#### Folivora: species >20 kg (all extinct)

**Figure 6:**
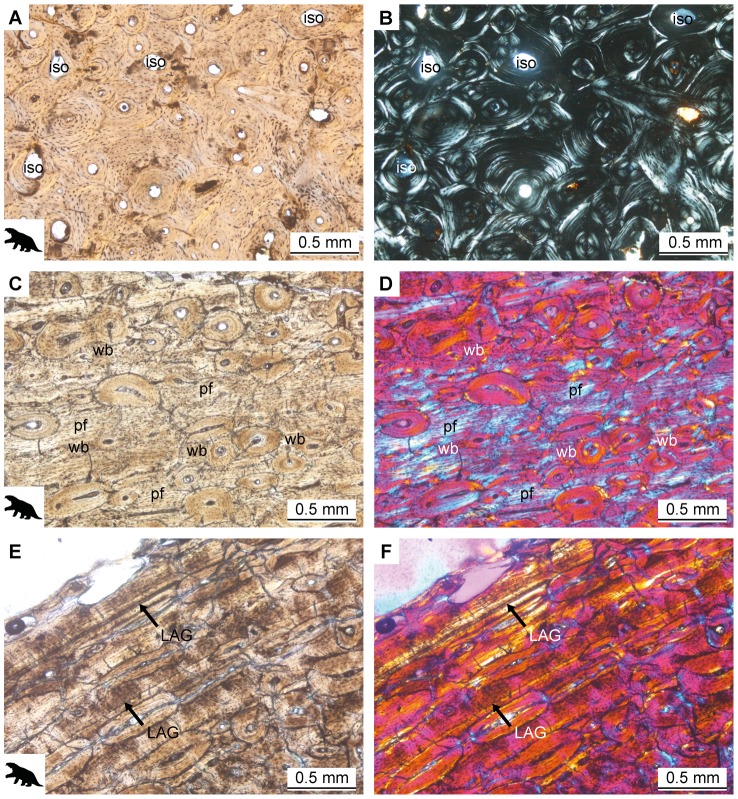
Bone histology of folivorans with a body mass larger than 20 kg. **A.** *Urumaquia robusta* AMU-CURS 169. Dense Haversian tissue of the femur with immature secondary osteons (iso) in normal light. **B.** The same as in A in cross-polarized light. **C.** Folivora indet. MHNSR-PV 1002. Secondarily remodeled matrix of woven, parallel-fibered and lamellar organization in the femur in normal light and **D.** in cross-polarized light with λ-compensator. **E.** *Mirandabradys zabasi* AMU-CURS 128. Random arrangement of secondary osteons and areas of lamellar bone (arrows) in the femur in normal light and **F.** in cross-polarized light with λ-compensator.

The cortices of large folivoran long bones are vascularized by randomly arranged canals with longitudinal orientation. Most canals are circular in section and vary in size. The bone tissue of the cortices is mainly composed of dense Haversian bone, with several generations of partly overlapping secondary osteons. These secondary osteons are clearly distinguishable under cross-polarized light due to their clear cement lines. Many of the fossil folivoran long bones sectioned show bone remodeling in progress, since some resorption cavities and many immature secondary osteons are present throughout the cortices (Figure 6a–b). In some cases, remains of the primary tissue are still visible, suggesting that it had been of parallel-fibered and lamellar organization. In the case of MHNSR-PV 1002, MHNSR-PV 1006 and MHNSR-PV 1007 the primary matrix is composed of a mixture of woven, parallel-fibered and lamellar tissue (Figure 6c–d). In areas of woven bone, osteocyte lacunae are round and randomly oriented, whereas in areas of parallel-fibered or lamellar bone osteocyte lacunae are elongated, flattened and arranged in a parallel manner. MHNSR-PV 1002 and MHNSR-PV 1006 also show strong laminar organization of the tissue in the exterior part of the cortex, with secondary osteons being elongated (Figure 6e–f). In this layer, two LAGs can be identified for MHNSR-PV 1006 (Figure 6e–f). The thin strips of bone lining the medulla and the trabeculae are composed of parallel-fibered and lamellar tissue. Trabeculae vary in thickness between samples but form a dense network in all elements sectioned.

**Figure 7:**
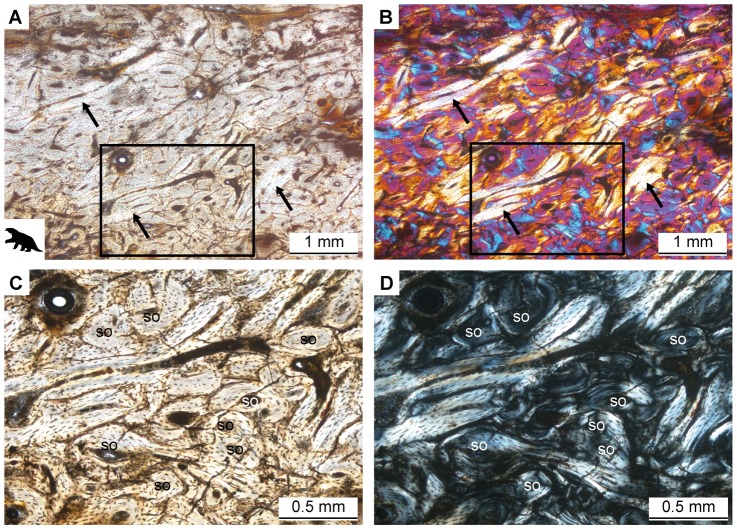
Histology of the femur of *Mirandabradys zabasi* AMU-CURS 128. Irregular arrangement of secondary osteons and areas of lamellar bone (arrows) representing secondary osteons with an oblique orientation within the three-dimensional bone tissue. **A.** In normal light and **B.** in cross-polarized light with λ-compensator. **C.** Close-up of area indicated by the black box in A and B showing overlapping generations of secondary osteons (so) in normal and **D.** in cross-polarized light. Note the flattened osteocyte lacunae typical of lamellar bone.

In *Mirandabradys zabasi* AMU-CURS 128, bone tissue organization varies along the mediolateral axis. Secondary osteons are oblong and overall tissue organization is somewhat laminated in the middle of the anterior and posterior parts of the cortex. More laterally, the laminated organization of the tissue is lost and many obliquely or longitudinally sectioned secondary osteons can be found (Figure 7a–b). The density of the Haversian tissue with overlapping generations of secondary osteons (Figure 7c–d) indicates that the entire primary bone tissue has been remodeled. Lamellar bone lines the cavities in the cancellous area of the lateral cortex. AMU-CURS 128 and *Megalocnus rodens* AMNH u2 also show that the folivoran femur is locally cancellous in the lateral region.

#### Folivora: species <20 kg (all extant)

**Figure 8:**
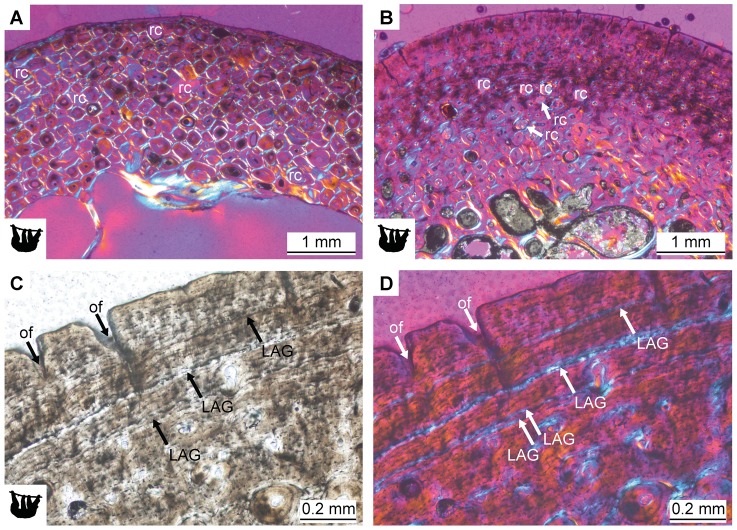
Histology of long bones of folivorans with a body mass smaller than 20 kg. **A.** Overview of the cortical bone tissue in the humerus of *Choloepus didactylus* ZMZ 17223. **B.** Overview of the cortical bone tissue in the humerus of *Bradypus torquatus* ZMZ 11102. **C.** Lines of arrested growth (LAGs) in the outer cortex of the humerus of *Bradypus tridactylus* NMB 10488 in normal light and **D.** in cross-polarized light with λ-compensator. Note the double LAG consisting of two very closely spaced LAGs visible in F. rc resorption cavities, of open foramina of radial canals.

Long bones of small-sized folivoran specimens possess thick compact cortices and relatively loose networks of thick trabeculae. Trabeculae and the thin strip of secondary bone lining the medulla are composed of lamellar bone. The cortices are vascularized by mostly longitudinally oriented canals forming primary osteons, and many radially oriented canals in the external layer. Vascular canals are small, but a few large secondary cavities are found in the external layers of the cortices. These larger cavities could represent resorption cavities, since cement lines surrounding them are visible under cross-polarized light. All specimens show heavily remodeled cortical bone tissue, but they differ in the extent of remodeling (Figure 8a–b). Cortices in bones of *Choloepus didactylus* ZMZ 17223 are entirely made up of very dense Haversian bone, showing bone remodeling in progress by the presence of large resorption cavities. Only a very thin layer of lamellar tissue lines the cortices externally. No LAGs can be identified in ZMZ 17223. Humeri and femora of *Bradypus torquatus* ZMZ 11102 and *Bradypus tridactylus* NMB 10488, on the other hand, show a thick layer of primary bone matrix in the external cortex. This layer of primary bone is of the parallel-fibered and lamellar type, with elongated and flattened osteocyte lacunae. Additionally, LAGs are identified in this layer. NMB 10488 shows two single LAGs and one double LAG in the humerus (Figure 8c–d) and up to six LAGs in the femur. The humerus of ZMZ 11102 shows two closely spaced LAGs near the outer edge of the bone, to which the femur additionally shows a more widely spaced third LAG towards the interior of the bone.

#### Vermilingua

**Figure 9:**
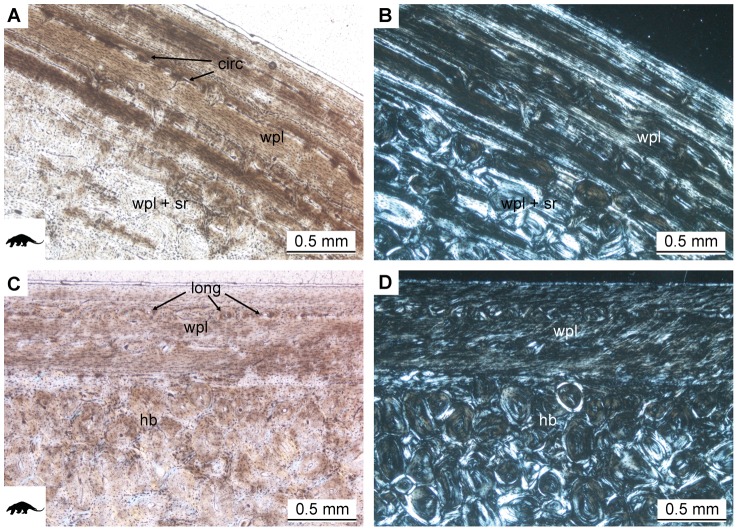
Histology of the outer cortex of the humerus of *Myrmecophaga tridactyla* ZMZ 11119. **A.** Close-up of region with circular vascular canals (circ) arranged in circular rows in normal light and **B.** in cross-polarized light. **C.** Close-up of region with longitudinal vascular canals (long) arranged in circular rows in normal and **D.** in cross-polarized light. hb Haversian bone, sr secondary remodeling, wpl tissue with woven, parallel and laminar organization of fibers.

The external cortices of anteater long bones are vascularized by circular, longitudinal and reticular canals of primary osteons, and these canals are arranged in concentric rows (Figure 9), thus resulting in a laminar organization. This laminar organization is most pronounced in *Tamandua tetradactyla* NMB 10420 in that vascular canals are larger than in the other two specimens of the same species (PIMUZ A/V 4807 and PIMUZ A/V 4808) and the bright lines between laminae are clearly visible. This does, however, not imply that NMB 10420 is older than the other two specimens of *T. tetradactyla*. On the contrary, the cortex of PIMUZ A/V 4807 shows more laminae than NMB 10420 and the bone tissue of PIMUZ A/V 4808 has been secondarily remodeled, both indicating that NMB 10420 represents a younger specimen. This is in accordance with the less advanced state of epiphyseal fusion and the smaller size of NMB 10420.

In the humeri and femora of *T. tetradactyla* NMB 10420 and *T. tetradactyla* PIMUZ A/V 4807, arrangement of vascular canals is random on the lateral side and radial on the medial side of the bone. The internal part of the cortex is vascularized by randomly arranged longitudinal canals. Trabeculae in the cancellous medullary region are thick in NMB 10420, PIMUZ A/V 4807 and *T. tetradactyla* PIMUZ A/V 4808, but fine in *Myrmecophaga tridactyla* ZMZ 11119. In all bones, the trabeculae form a dense meshwork throughout the medulla.

The tissue in the cortices of anteater long bones is mainly composed of a mixture of woven, parallel-fibered and lamellar bone conforming to the arrangement of the vascular canals (Figure 9). Up to five LAGs can be identified in the femur of ZMZ 11119, whereas the humerus only shows one distinct LAG. NMB 10420 shows no LAGs, but PIMUZ A/V 4807 and PIMUZ A/V 4808 show one LAG in the humerus and two LAGs in both humerus and femur, respectively. As already indicated above, secondary remodeling is not advanced in NMB 10420 and PIMUZ A/V 4807, and the few secondary osteons formed are mainly found in the medial and lateral regions of the bone. In PIMUZ A/V 4808, secondary remodeling is more advanced, with scattered secondary osteons occurring throughout the internal cortices. The internal layer of the cortices of ZMZ 11119 is heavily remodeled (Figure 9), but patches of primary bone matrix are still visible between secondary osteons. Trabeculae are composed of parallel-fibered and lamellar bone tissue.

### Bone Compactness Analysis

#### Bone global compactness of xenarthran humeri and femora

**Figure 10:**
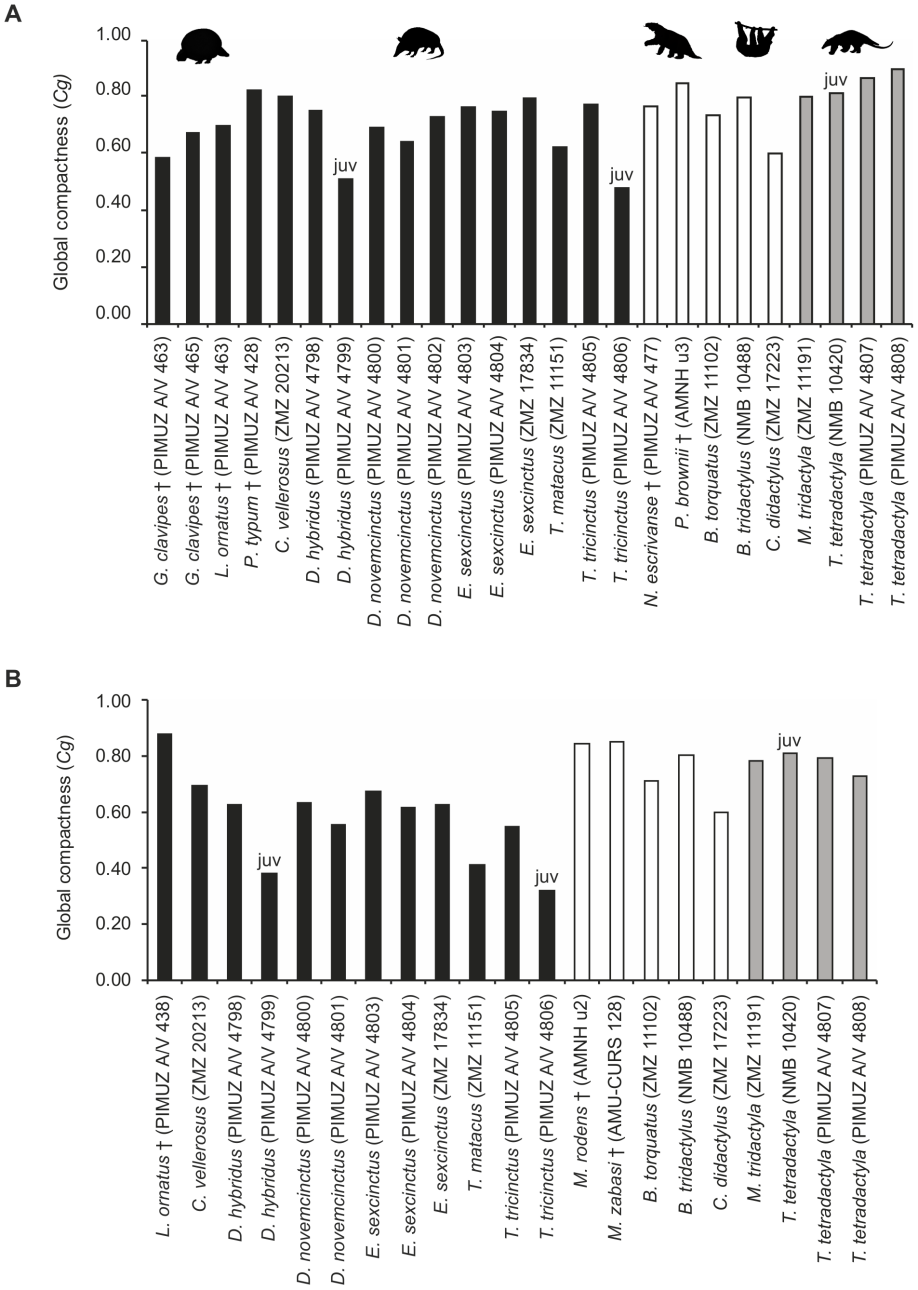
Bone global compactness (*Cg*) values of humeri (a) and femora (b) included in this study. Black bars represent members of Cingulata, white bars members of Folivora, and grey bars represent members of Vermilingua. Values for extant taxa are based on the average *Cg* values of two thin sections produced from the same bone. Juvenile specimens are marked with ‘juv’.

Bone global compactness (Cg) is highly variable across the whole group, within subgroups and also between individuals of the same species (Figure 10, Tables S2.1 and S2.2). The variation found in this study is further increased by the inclusion of the three juvenile specimens in this study. However, upon excluding these specimens, values still range between 0.600 (ZMZ 17223) and 0.879 (PIMUZ A/V 4808) for the humerus, and between 0.412 (ZMZ 11151) and 0.877 (PIMUZ A/V 438) for the femur. All adult specimens show a *Cg* value greater than 0.400, indicating that ≥40% of the cross-sectional area is composed of dense mineralized tissue. *Cg* values for anteater humeri are on average found to be slightly higher than those found for humeri of Cingulata and Folivora. However, considering the overall pattern of variation, this could be an artifact of the small sample size of Vermilingua. Furthermore, this pattern is no longer found when values are plotted according to species instead of individuals. In general, no distinct patterns in relation to phylogeny, body size or locomotion can be detected among *Cg* values for humeri of the studied xenarthrans.

The *Cg* values found for xenarthran femora do not suggest any striking differences between categories of body size or locomotion types either. However, the femora of sloths and anteaters show higher average values than those of cingulates. The same pattern is also found when plotting values according to species instead of individuals. Considering that sloths and anteaters form the monophyletic clade Pilosa, this may suggest a phylogenetic pattern for bone compactness in the femur.

**Figure 11:**
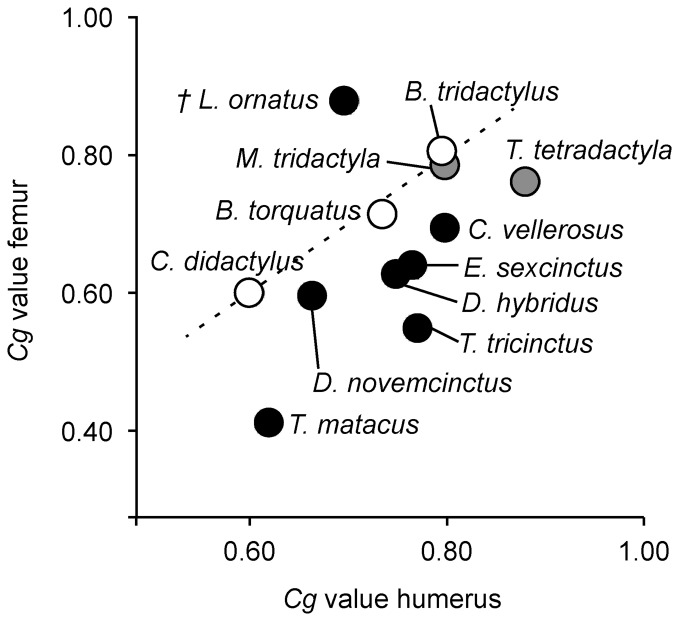
Average bone global compactness (*Cg*) of the humerus plotted against *Cg* values of the femur. Values were plotted for xenarthran species for which both bones were available. Black circles represent members of Cingulata, empty circles represent members of Folivora, and grey circles represent members of Vermilingua. The identity line is indicated (dashed line).

A plot of *Cg* values of humeri and femora from individuals for which both bones were available is given in Figure 11. This group includes one fossil species (*Lomaphorus ornatus*). The distribution of the data points reflects the variation detected when plotting values for the humerus and the femur individually (Figure 10). Overall, a positive correlation is found between the *Cg* of the humerus and the *Cg* of the femur, suggesting that these long bones do not represent independent modules. The data for extant sloths and for *Myrmecophaga tridactyla* falls on the identity line, suggesting that humeral *Cg* and femoral *Cg* in these species are equal. Data points for armadillos and *Tamandua tetradactyla*, on the other hand, are found below the identity line, suggesting an allometric relationship, with *Cg* in the humerus being higher than in the femur. *Lomaphorus ornatus* is the only species that plots above the identity line, showing a higher compactness in the femur than in the humerus.

#### *P* and *S* values of adult xenarthran humeri

**Figure 12:**
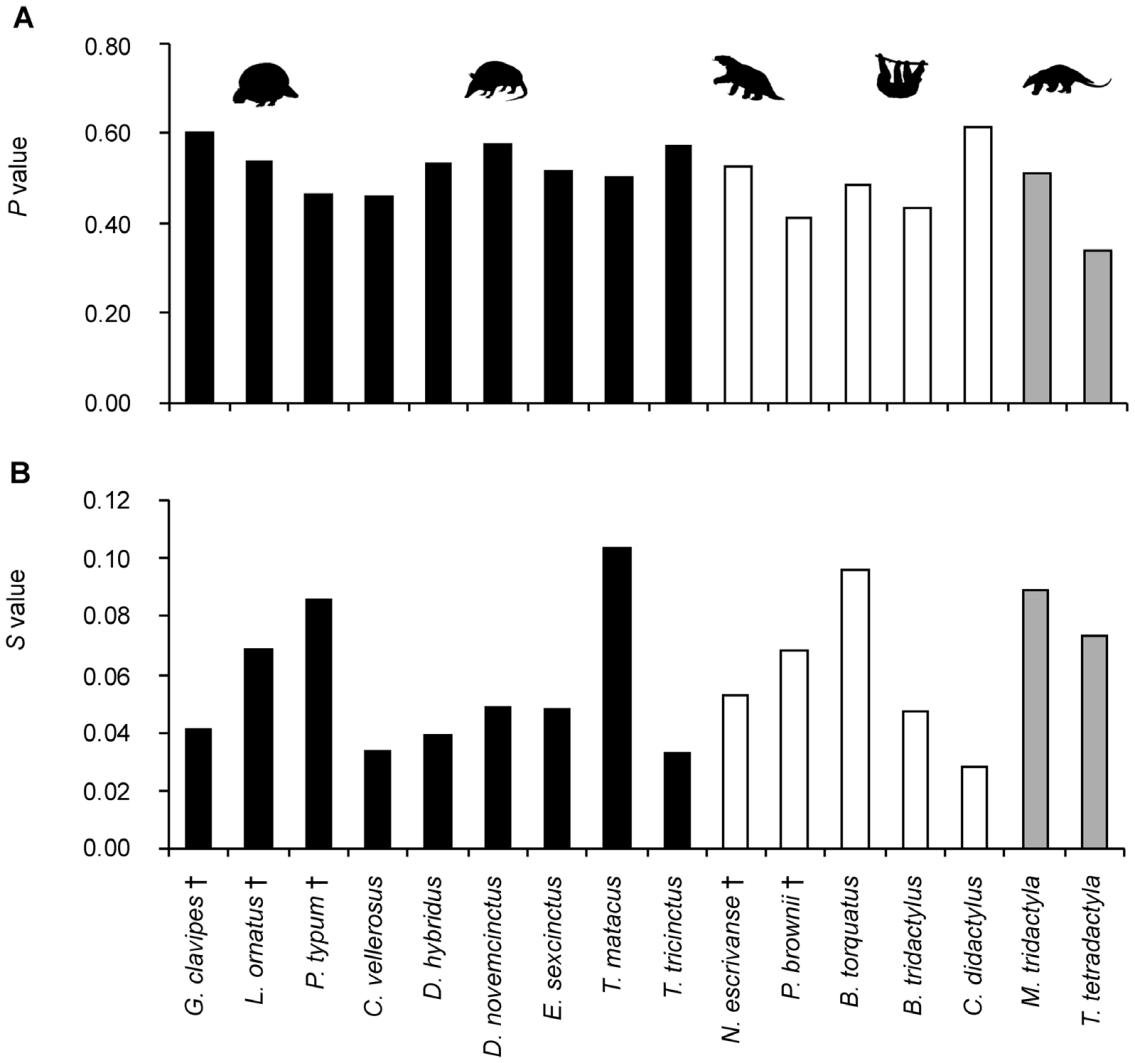
P and S values for humeri of species included in this study. **A.** Relative distance from the section center to the inflexion point (*P*). **B.** Reciprocal of the slope at the inflexion point (*S*). All values are based on bones of adult individuals only. Values for extant taxa represent average *Cg* values of at least two thin sections produced from the same bone or from bones of different individuals. Black bars represent members of Cingulata, white bars members of Folivora, and grey bars represent members of Vermilingua.

Bone Profiler values for the relative distance from the section center to the inflexion point (*P*) and for the reciprocal of the slope at the inflexion point (*S*) [16] in xenarthran humeri do not show any distinct patterns related to phylogeny, body size or locomotion (Figure 12; for exact values see Table S2.1). Variation of *P* values is moderate across Xenarthra, as well as within Cingulata, Pilosa and Vermilingua. Values for *S*, on the other hand, show extensive variation across Xenarthra, both within the three subgroups and within genera, signifying that the transition from the medulla to the compact cortex is gradual in some and abrupt in other taxa. No pattern related to phylogeny, body size or locomotion can be detected.

#### *P* and *S* values of adult xenarthran femora

**Figure 13:**
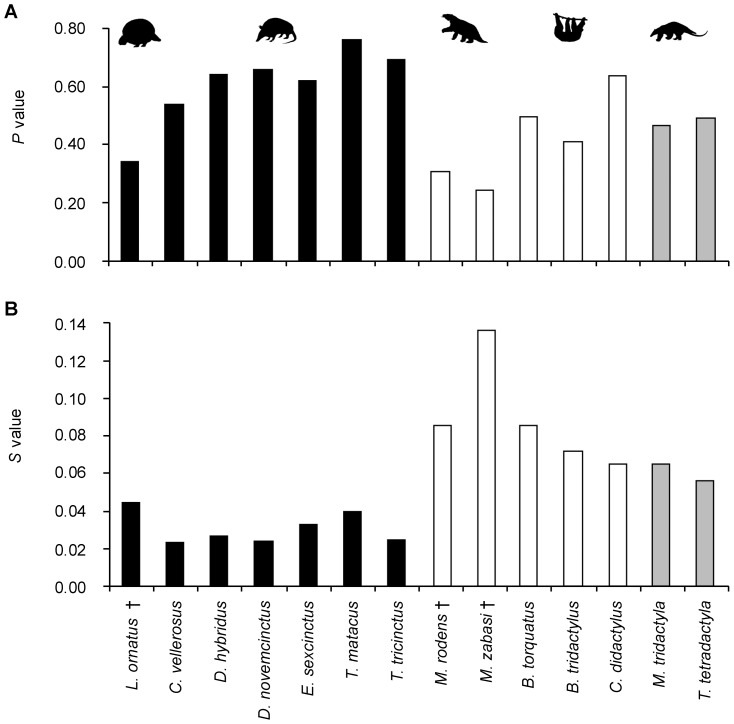
P and S values for femora of species included in this study. **A.** Relative distance from the section center to the inflexion point (*P*). **B.** Reciprocal of the slope at the inflexion point (*S*). All values are based on bones of adult individuals only. Values for extant taxa represent average *Cg* values of at least two thin sections produced from the same bone or from bones of different individuals. Black bars represent members of Cingulata, white bars members of Folivora, and grey bars represent members of Vermilingua.

Values for both *P* and *S* of the femur are variable within the three subgroups Cingulata, Folivora and Vermilingua, and within genera contained therein (Figure 13, for exact values see Table S2.2). However, *P* values are on average higher for femora of members belonging to Cingulata than for members of either of the two groups within the sister clade Pilosa. This signifies that the relative distance from the center of the bone to the transition point from cancellous medullary region to cortical compacta is greater in Cingulata than in Folivora and Vermilingua. In terms of cross-sectional geometry, this indicates that the compact cortex of the cingulate femur occupies a smaller proportion of the cross-sectional area than the cortex of a pilosan femur. This is consistent with the slightly lower average value of *Cg* found for Cingulata in comparison with the average values found for Pilosa. The *S* values show a reverse and more distinct pattern, with the average value for Pilosa being higher than the average value for Cingulata. The transition from a relatively cancellous medullary region to a relatively compact cortex is therefore more abrupt in cingulate femora than in folivoran or vermilinguan femora. In summary, these results suggest that a phylogenetic pattern is contained in *P* and *S* values for the femora.

#### Ontogenetic changes in bone compactness

**Figure 14:**
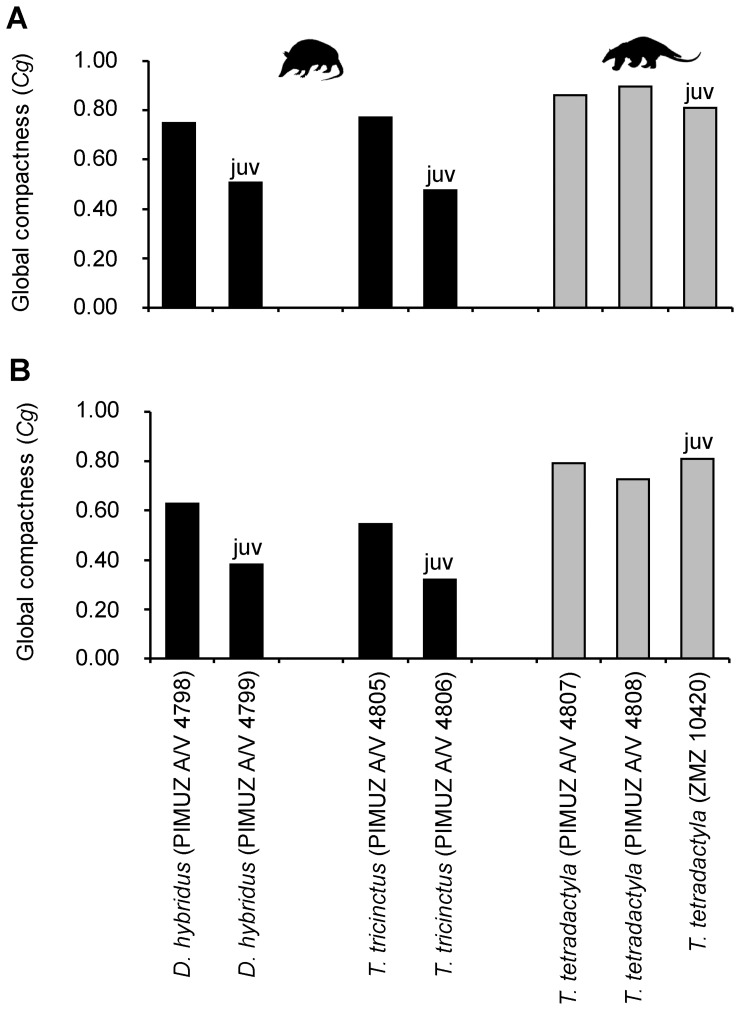
Comparison of *Cg* values between adult and juvenile specimens. **A.** Values for humeri and **B.** values for femora of species for which both juvenile and adult specimens were available. Juvenile specimens are marked with ‘juv’.

Figure 14 shows a comparison of *Cg* values for adult and juvenile specimens of *Dasypus hybridus*, *Tolypeutes tricinctus,* and *Tamandua tetradactyla.* In armadillos, *Cg* values of juveniles are found to be smaller than *Cg* values of adults for both humerus and femur. In *Dasypus hybridus*, juvenile *Cg* for humeri amounts to 67.8% of the adult *Cg* value, while in *Tolypeutes tricinctus* it amounts to 61.9% of the adult *Cg*. For femora, juvenile *Cg* values represent 60.9% and 58.3% of the adult *Cg* value respectively. These results thus indicate that *Cg* in armadillo long bones increases extensively during development. In the anteater *Tamandua tetradactyla* juvenile and adult *Cg* values differ by a maximum of about 11% for both bones. The *Cg* value of the juvenile specimen is lower than both adult *Cg* values in the humerus and higher than both adult *Cg* values in the femur. This suggests that bone compactness in the humerus of *Tamandua tetradactyla* increases during development, whereas compactness of the femur decreases. However, the ontogenetic series for *Dasypus hybridus*, *Tolypeutes tricinctus* and *Tamandua tetradactyla* each include two growth stages only. A thorough investigation into ontogenetic changes in bone compactness of xenarthran long bones would be worthwhile in order to test whether the pattern found for *T. tetradactyla* is not just a product of chance but applies to vermilinguans in general, or even to all members of Pilosa.

## Discussion

### Xenarthrans in the Context of Mammalian Bone Histology

Xenarthrans possess a long bone microstructure consistent with other placental mammals in general [2], [5], [21], [22], [24], [25], [73]–[77]. Table 3 summarizes histological features described for long bones of four clades of placental mammals. Although the descriptions for some of these clades are based on limited sampling, this summary shows hypothetical patterns that can be further investigated. In terms of vascularization, xenarthran long bones show a pattern more similar to that of Euarchontoglires. Vascular canals are oriented longitudinally, reticularly or radially and they are arranged in an irregular manner. Only anteater long bones exhibit areas with a circular arrangement of vascular canals. According to the histological descriptions summarized in table 3 [5], [22], [24], [25], [76], [77] members of Laurasiatheria and Afrotheria, on the other hand, often possess circumferential vascular canals and a laminar to plexiform vascular network. The primary bone tissue of xenarthran long bones comprises a mixture of woven, parallel-fibered and lamellar bone and is thus more similar to that of Laurasiatheria and Afrotheria than to the primary bone tissue of Euarchontoglires. The latter can be fully lamellar or of the woven and parallel-fibered type. However, considering that the available data on the bone histology of Euarchontoglires is limited and that a mixed bone tissue (woven plus lamellar and/or parallel-fibered bone) was found in bones of stem eutherians [75], the bone tissue of Euarchontoglires is probably not so different from that of the remaining clades of placental mammals. Haversian bone has been found in all four clades of placental mammals. Xenarthra is, however, the only clade in which all investigated subgroups show this heavily remodeled bone tissue.

**Table 3:** Generalized pattern of histological long bone features among major clades of placental mammals.

Histological features	Euarchontoglires	Laurasiatheria	Afrotheria	Xenarthra
***Orientation of vascular canals***	longitudinal, reticular, radial	circumferential, longitudinal, reticular, radial	circumferential, longitudinal,reticular	longitudinal, reticular, radial
***Pattern of vascularization***	irregular	irregular *or* laminar to plexiform	laminar to plexiform	irregular *and/or* circular
***Primary bone***	lamellar *or* woven and parallel-fibered	woven, parallel-fibered andlamellar	woven, parallel-fibered andlamellar	woven, parallel-fibered and lamellar
***LAGs***	yes	yes	yes	yes
***Remodeling***	Haversian bone, except in rodents	Haversian bone, except insmall animals	Haversian bone, except insmall animals	Haversian bone

Feature descriptions for Euarchontoglires, Laurasiatheria and Afrotheria were adapted from [5], [22], [24], [25], [72]–[78], [99].

Information on the presence of LAGs within placental mammals is incomplete, but LAGs have been documented for at least some members of all four major clades (Table 3). Zonal bone tissue, indicating periodically interrupted deposition of bone during later ontogeny, has been documented for marine mammals (e.g. cetaceans and pinnipeds [78]), mammals of temperate regions [78], hibernators [75], [78], polar bears [72], and ruminants from a variety of environments [25]. Additionally, Curtin et al. [77] investigated bone microstructure in fossil and extant neonatal elephantids and found hypercalcified lines of arrested osteogenesis in their femora and tibiae. The extinct Plio-Pleistocene bovid *Myotragus* [76] also showed zonal deposition of bone tissue, and Köhler and Moyà-Solà [76] argued that interrupted growth in *Myotragus* could have been an adaptive strategy in a resource-limited environment. Sander and Andrássy [24] found LAGs in the large majority of their sample of large fossil mammals from Germany and thus proposed that LAGs might be a universal feature of large mammals. Indeed, flexible growth rates, indicated by the deposition of LAGs, are suggested to be plesiomorphic to the mammalian lineage and could have played a major role in the diversification pattern of mammals after the K/T extinction [75]. Hurum and Chinsamy [75] further suggested that eutherian mammals developed rapid, uninterrupted growth during the Paleocene, spurring an increase in body size, and that modern eutherians retained the plesiomorphic condition of interrupted growth (see also Chinsamy and Hurum [74]). Moreover, a recent study on seasonal growth in ruminants suggests that flexible growth is a universal feature of homeothermic endotherms [25].

The studied sample of long bones confirms that flexible growth rates, and thus LAGs, are common in Xenarthra. All extant specimens representing skeletally mature adults, i.e. those with fused epiphyses, show LAGs in the cortices of their long bones. The only exception to this is *Choloepus didactylus* ZMZ 17223, which is interpreted to be a specimen of advanced age since its bone microstructure is completely remodeled. In adult armadillo long bones the occurrence of LAGs is restricted to the outermost layer of the cortex, thus representing an OCL or EFS. In bones of anteaters and sloths, LAGs are not restricted to this outer layer but are also found within the middle part of the cortex, provided that the cortex is not completely remodeled. This pattern can be explained by taking the age at which these species reach sexual maturity into consideration. Most mammals reach sexual maturity shortly after achieving skeletal maturity, i.e. adult size [79], but slow circumferential periosteal growth of the bones in diameter, including the deposition of closely spaced LAGs, can continue beyond this point [80]. The extant folivoran taxa included in this study represent species that reach sexual maturity after two or more years [81], [82]. Thus it is no surprise that the specimens assigned to these species show LAGs both in the middle part of the cortex and towards the periphery, as long as they are not older adults as is the case of *Choloepus didactylus* ZMZ 17223. The extant species of cingulates included in this study all reach sexual maturity within or shortly after their first year [83] and a clear line separates the inner remodeled part of the cortex from the thin outer parallel-fibered or lamellar layer in adult specimens. This line could mark the attainment of sexual maturity, after which bone tissue was only very slowly deposited and LAGs are closely spaced.

The presence of LAGs in MHNSR-PV 1006 raises the question of whether LAGs were formed in other fossil specimens but are simply masked by extensive secondary remodeling. The presence or absence of LAGs could provide information on the attainment of sexual maturity and the growth dynamics in extinct taxa. Since MHNSR-PV 1006 represents a pilosan taxon it can be assumed that LAGs are also common among extinct sloths and anteaters, and that these taxa probably reached sexual maturity after two or more years. Considering the size of glyptodonts and pampatheriids compared to their extant relatives, it would be of interest to find out whether they reached sexual maturity at about the same age or later than modern cingulates. Wolf [39], [56] and Hill [52] found surface-parallel growth marks, in some cases possibly representing true LAGs, in the superficial layer of the cortex of most of the fossil osteoderms sampled. It is unclear, however, how growth of osteoderms relates to growth of long bones in these animals.

### Secondary Remodeling and Size

The cortices of xenarthran long bones are well vascularized and often heavily remodeled. Enlow and Brown [5] noted that dense Haversian bone is a common feature found in mammalian bones, but is absent in some groups such as monotremes, marsupials, eulipotyphlans and rodents. The thin sections produced for this study show that secondary remodeling is found in the long bones of all xenarthrans. Armadillos exhibit a special type of Haversian bone in that certain areas show irregularly shaped secondary osteons. Among extant xenarthrans, sloths show the highest extent of secondary remodeling, and only fossil forms show a comparable extension of Haversian bone in the cortex.

The histology of cingulate and folivoran long bones included in this study differed between large (>20 kg) and small (<20 kg) taxa. It is noteworthy that for folivorans and cingulates included in this study, these body mass categories also correspond to the extinct/extant status of each taxon; i.e., large taxa represent extinct forms and small taxa represent extant forms. Secondary remodeling in bones of fossil cingulates and folivorans was much more extensive than in those of extant species, with the exception of the bones of *Choloepus didactylus* ZMZ 17223. Although the microstructure of fossil bones was greatly masked by diagenetic alteration of the tissue, it was still possible to detect dense Haversian bone that most likely constituted the entire cortices of these bones. The extent of secondary bone remodeling could be a feature in which extinct (and thus large) cingulates and folivorans differ from their extant (and thus small) relatives. A possible scenario is that extinct forms of Cingulata and Folivora lived longer than extant forms. A prolonged life span could also explain differences in size between extinct and extant xenarthrans, and increased size would have resulted in increased loading and consequently more remodeling in the larger (extinct) forms. On the other hand, the observed pattern could also be a result of chance, i.e. fossil specimens and *Choloepus didactylus* ZMZ 17223 represent more mature individuals than all remaining extant specimens. However, because we possess no data on the chronological age of fossil and extant specimens included in this study, other than that the epiphyses are fused in all but three specimens (PIMUZ A/V 4799, PIMUZ A/V 4806 and NMB 10420), this question remains unanswered. Bone histology in an ontogenetic series of fossil and extant xenarthran taxa could possibly reveal whether differences in the rate of secondary remodeling or the life span of extinct and extant taxa really exist.

### Bone Compactness Parameters

The parameters *Cg* (global compactness), *S* (reciprocal of slope at inflexion point) and *P* (relative distance from center of bone to inflexion point) of the bone compactness profiles of xenarthran long bones show no ecological signal of the sort recently detected in humeri of a large sample of amniotes [13], a signal apparently lacking in talpid moles [20]. In fact, the compactness parameters of xenarthran humeri show neither a pattern related to locomotion or body size, nor a pattern related to phylogeny. However, the structural component sensu Cubo et al. [7] was not investigated in this study. A pattern possibly related to phylogeny is found for all three parameters of xenarthran femora. Phylogenetic signal has also been detected in these parameters for lissamphibian long bones [17] and in the radius of amniotes [10]. *Cg* and *S* values for femora of members of Pilosa are higher than values found for members of Cingulata. For *P* values, an inverse relationship is found with values being higher in Cingulata than in Pilosa. These results suggest a phylogenetic pattern in the bone compactness profile of the femur and also show that the parameters *Cg*, *S* and *P* are linked. The average *S* values for cingulate femora, for example, are lower than the *S* values found for Pilosa when *Cg* values of cingulate femora are lower than *Cg* values found for Pilosa. A negative relationship is found between values of *P* and *Cg*. This linkage of parameters indicates that not only the thickness of the compacta but also the transition (abrupt vs. gradual) from medulla to cortex have a direct influence on bone compactness.

### Allometry in Bone Compactness

**Figure 15:**
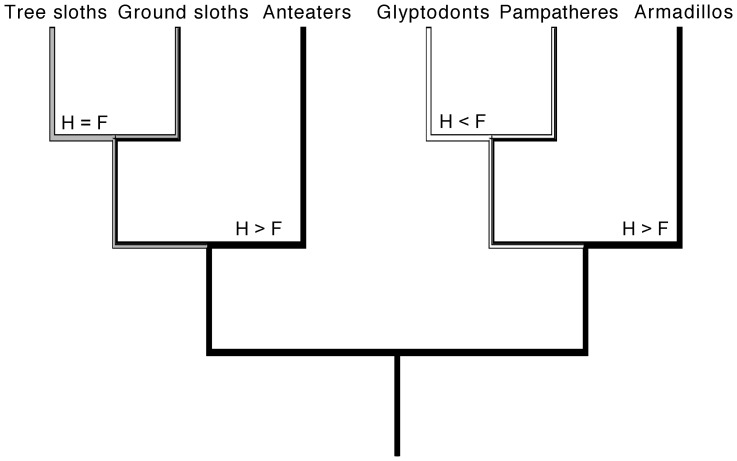
Traced history of the relationship between humeral (H) and femoral (F) bone compactness. History of humeral and femoral bone compactness was traced for the main groups of Xenarthra. Note that the state for anteaters is set to H>F, because this state is displayed by the majority of the samples included in this study.

The pattern found in the relationship between humeral and femoral bone compactness in xenarthrans could possibly be related to locomotion and life habit. Figure 15 shows the traced history of the relationship between humeral and femoral bone compactness. The 1∶1 relationship between bone compactness in the humerus and femur in modern sloths can be explained by the suspensory arboreality of these xenarthrans. In locomotory terms, the forelimb and the hind limb are equally important [84], [85] and therefore one could assume that they bear the same amount of stress, which then induces a more or less equal compactness in the humerus and femur. Armadillos, on the other hand, are scratch diggers [86] and show many specializations for digging in their forelimbs. They possess large claws, tuberosities for strong muscular insertions and long lever arms for the line of action of the principal muscles involved in digging [87]. Indices of the armadillo forelimb, for example the index of fossorial ability (IFA), have shown to correlate well with digging, while indices of the hind limb are linked to body size [87], [88]. The allometric relationship between humeral and femoral compactness found in this study, whereby humeral compactness is higher, is therefore most likely linked to digging and the associated morphological adaptations found in the humerus. Only members of Tolypeutini were noted to be non-diggers (e.g. [89]).

In the analysis of humeral and femoral compactness, *Lomaphorus ornatus* plotted as an outlier to all other cingulates (Figure 11). Femoral compactness in this glyptodont was much higher than the compactness of the humerus. The humeri of glyptodonts have been shown to lack features that would indicate digging ability [90], and Quintana [91] noted that the general anatomy of these extinct xenarthrans prevented them from a fossorial habit. The allometric relationship between humeral and femoral bone compactness of the glyptodont *L. ornatus* found in this study fits with this scenario.

The position of the two anteaters *Myrmecophaga tridactyla* and *Tamandua tetradactyla* in Figure 11 question the proposed relationship of relative humeral and femoral bone compactness with locomotion and life habit in xenarthrans. *M. tridactyla* plotted along the same line as sloths, whereas the data point for *T. tetradactyla* lies on the same line as digging armadillos. Anteaters are generally considered hook-and-pull diggers [86] that apply their specialized digging method for obtaining food rather than constructing burrows [92]. *M. tridactyla*, the giant anteater, exhibits an almost wholly terrestrial habit, whereas *T. tetradactyla* moves freely between terrestrial and arboreal environments [93]. It would therefore fit better with the proposed locomotion-related pattern if *M. tridactyla* plotted along with digging armadillos and *T. tetradactyla* with modern sloths. Including the almost fully arboreal silky anteater (*Cyclopes didactylus*) in future studies could possibly shed more light on whether relative compactness of the humerus and of the femur in xenarthrans is indeed linked to locomotion. In this context it would also be important to include mylodontid ground sloths such as *Scelidotherium* and *Glossotherium,* as limb bone proportions and resistance to bending forces suggest that these taxa were capable of performing strenuous activities like digging [94], [95]. Rare taxa such as the giant armadillo (*Priodontes*) or the extinct aquatic sloth *Thalassocnus* are difficult to obtain for histological analysis but might also reveal specialized patterns.

## Supporting Information

Figure S1 **Figure S1.1.** Binary images of thin sections produced from fossil xenarthran long bones. Catalogue numbers are provided in the respective order. **(a–f)** Humeri. (a–b) *Glyptodon clavipes* PIMUZ A/V 463 and PIMUZ A/V 465. (c) *Lomaphorus ornatus* PIMUZ A/V 438. (d) *Pampatherium typum* PIMUZ A/V 428. (e) *Nothrotherium escrivanse* PIMUZ A/V 477. (f) *Parocnus brownii* AMNH u3. **(g–i)** Femora. (g) *Lomaphorus ornatus* PIMUZ A/V 438. (h) *Megalocnus rodens* AMNH u2. (i) *Mirandabradys zabasi* AMU-CURS 128. **Figure S1.2.** Binary images of thin sections produced from armadillo humeri. Catalogue numbers are provided in the respective order. **(a)** *Chaetophractus vellerosus* ZMZ 20213. **(b–c)** *Dasypus hybridus* PIMUZ A/V 4798 and PIMUZ A/V 4799. **(d–f)** *Dasypus novemcinctus* PIMUZ A/V 4800, PIMUZ A/V 4801 and PIMUZ A/V 4802. **(g–i)** *Euphractus sexcinctus* ZMZ 17834, PIMUZ A/V 4803 and PIMUZ A/V 4804. **(j)** *Tolypeutes matacus* ZMZ 11151. **(k–l)** *Tolypeutes tricinctus* PIMUZ A/V 4805 and PIMUZ A/V 4806. **Figure S1.3.** Binary images of thin sections produced from armadillo femora. Catalogue numbers are provided in the respective order. **(a)** *Chaetophractus vellerosus* ZMZ 20213. **(b–c)** *Dasypus hybridus* PIMUZ A/V 4798 and PIMUZ A/V 4799. **(d–e)** *Dasypus novemcinctus* PIMUZ A/V 4800, PIMUZ A/V 4801. **(f–h)** *Euphractus sexcinctus* ZMZ 17834, PIMUZ A/V 4803 and PIMUZ A/V 4804. **(i)** *Tolypeutes matacus* ZMZ 11151. **(j–k)** *Tolypeutes tricinctus* PIMUZ A/V 4805 and PIMUZ A/V 4806. **Figure S1.4.** Binary images of thin sections produced from extant folivoran humeri. Catalogue numbers are provided in the respective order. **(a)** *Bradypus torquatus* ZMZ 11102. **(b)** *Bradypus tridactylus* NMB 10488. **(c)** *Choloepus didactylus* ZMZ 17223. **(d–f)** *Tamandua tetradactyla* NMB 10420, PIMUZ A/V 4807 and PIMUZ A/V 4808. **(g)** *Myrmecophaga tridactyla* ZMZ 11119. **Figure S1.5.** Binary images of thin sections produced from extant folivoran femora. Catalogue numbers are provided in the respective order. **(a)** *Bradypus torquatus* ZMZ 11102. **(b)** *Bradypus tridactylus* NMB 10488. **(c)** *Choloepus didactylus* ZMZ 17223. **(d–f)** *Tamandua tetradactyla* NMB 10420, PIMUZ A/V 4807 and PIMUZ A/V 4808. **(g)** *Myrmecophaga tridactyla* ZMZ 11119.
(PDF)

Table S1 List of samples and their specifications.
(XLS)

Table S2 **Table S2.1.** Bone profiler outputs for humeri included in this study. **Table S2.2.** Bone profiler outputs for femora included in this study.
(XLS)
