# Supplementary figures and images for: Evolutionary Patterns of Bone Histology and Bone Compactness in Xenarthran Mammal Long Bones
Source: PLoS One. 2013 Jul 9;8(7):e69275. doi: 10.1371/journal.pone.0069275 (PMC3706384; doi:10.1371/journal.pone.0069275)

a.

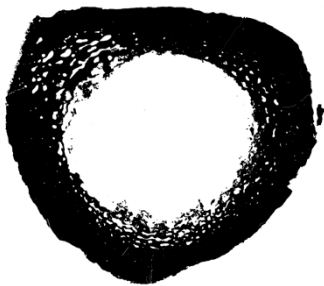

b.

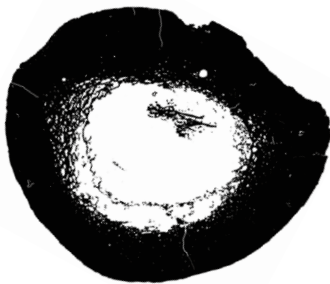

c.

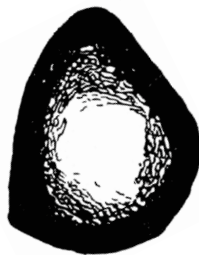

d.

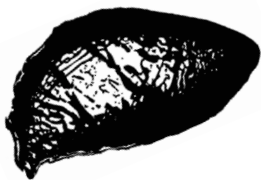

e.

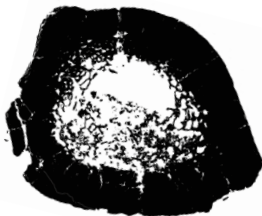

f.

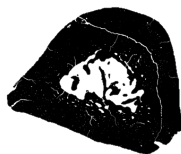

g.

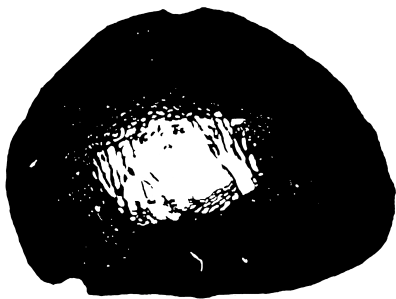

h.

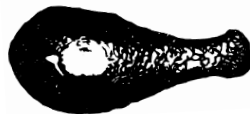

i.

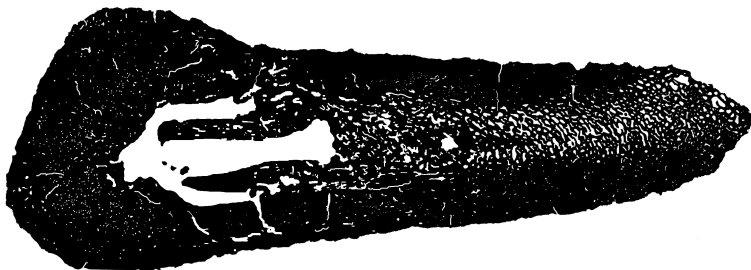

2 cm

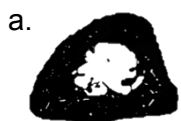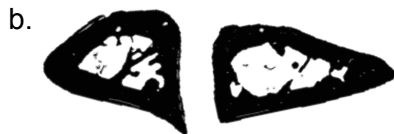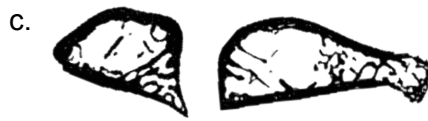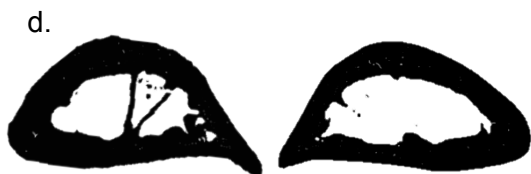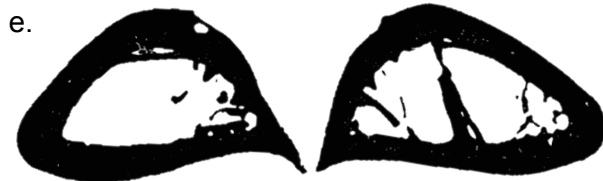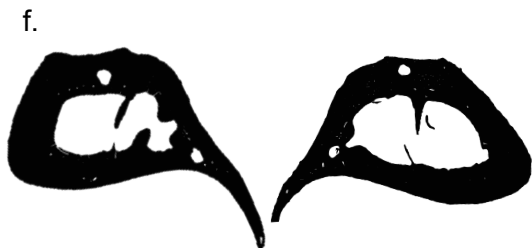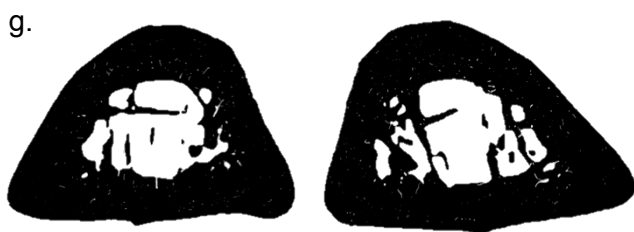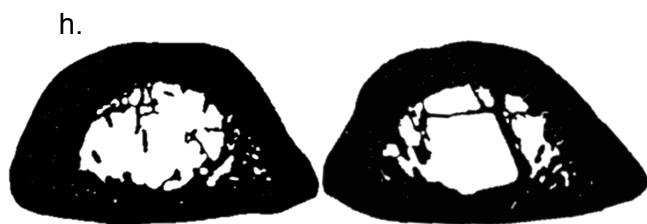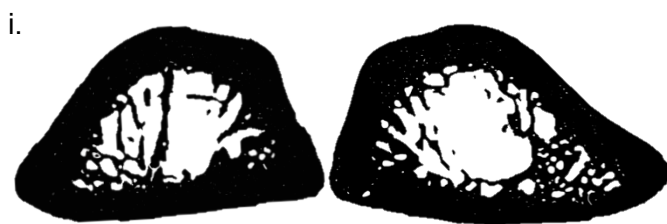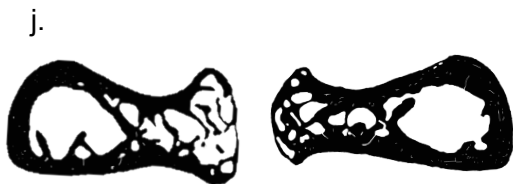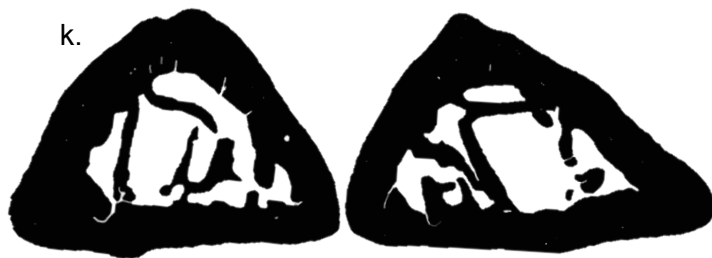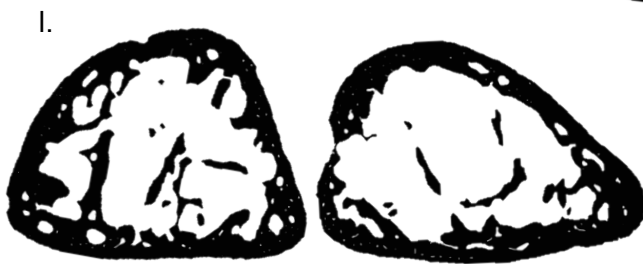

5 mm

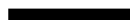

a.

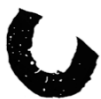

b.

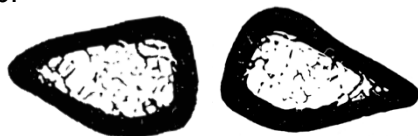

c.

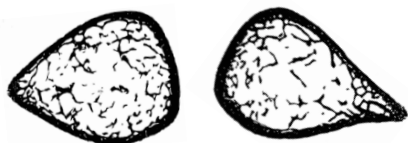

d.

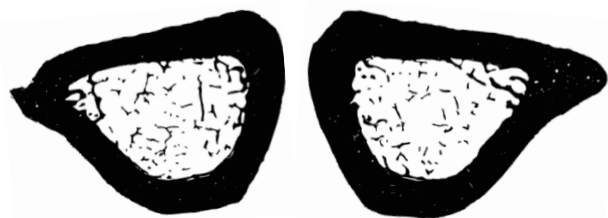

e.

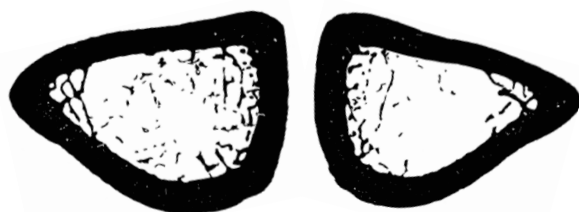

f.

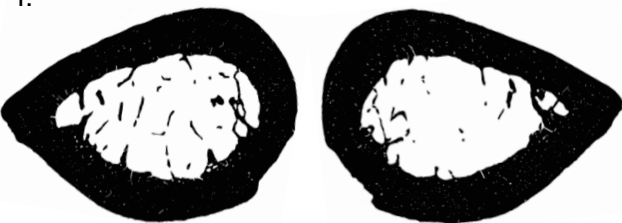

g.

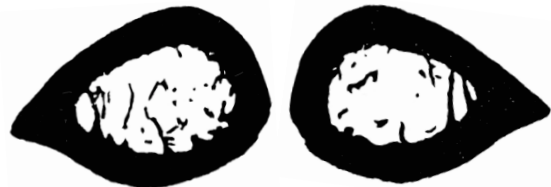

h.

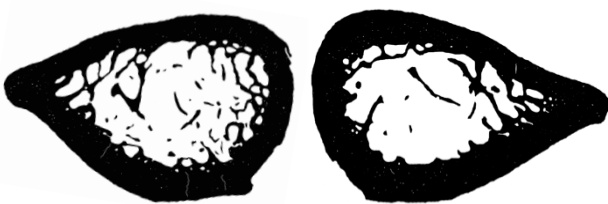

i.

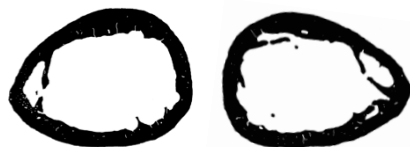

j.

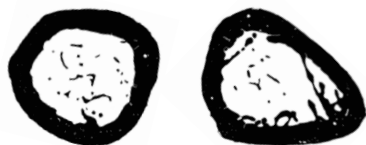

k.

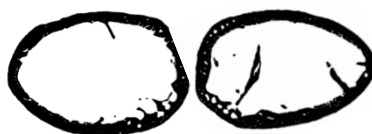

5 mm

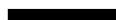

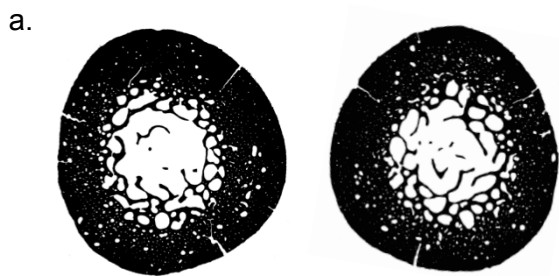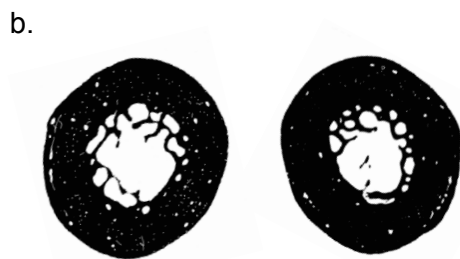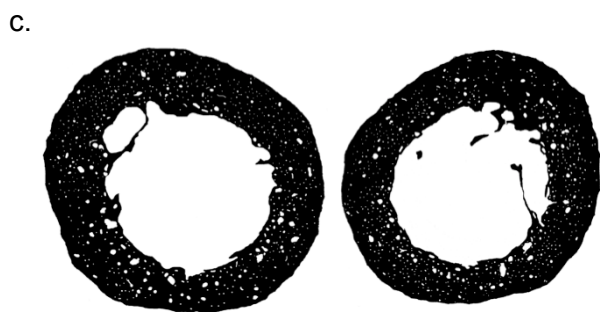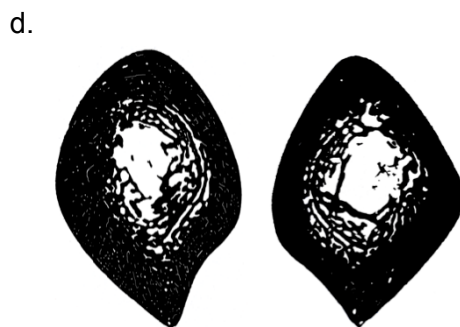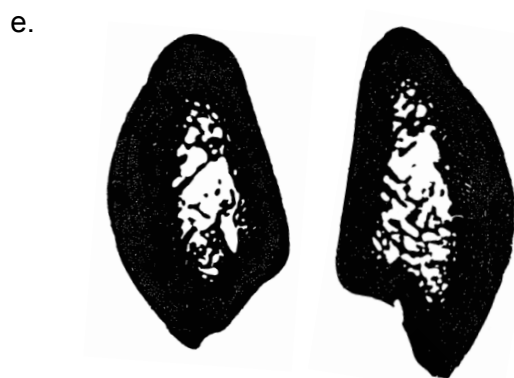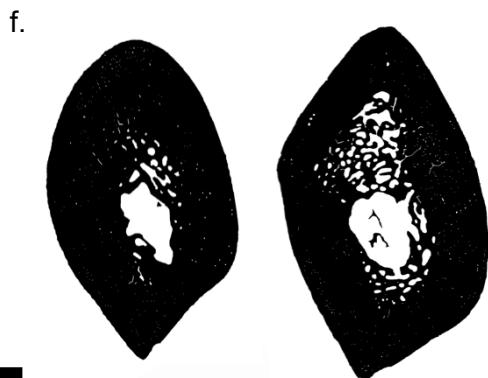

5 mm

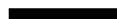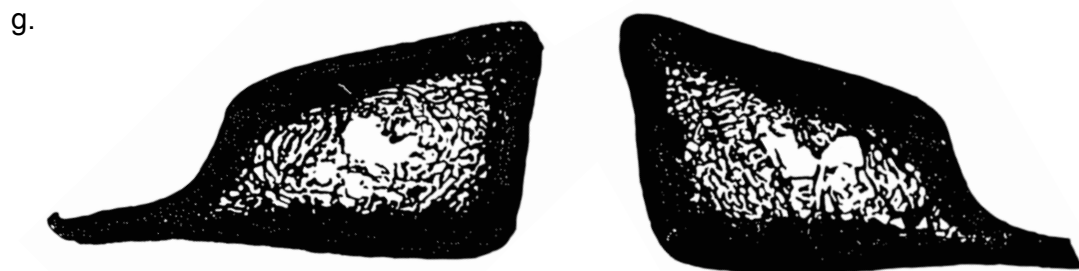

1.5 cm

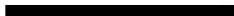

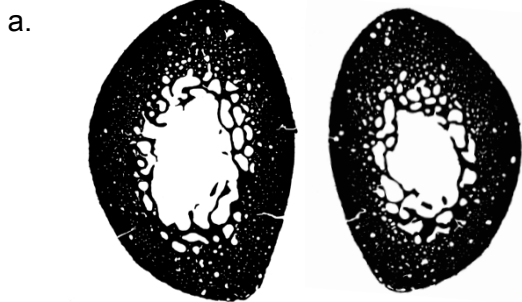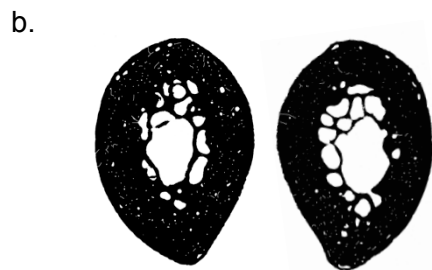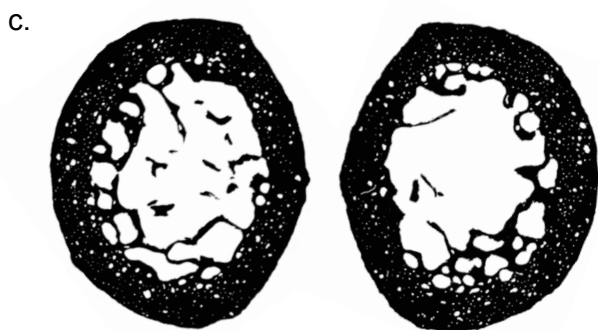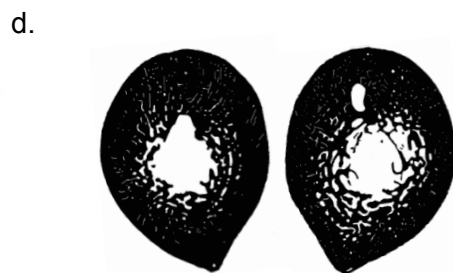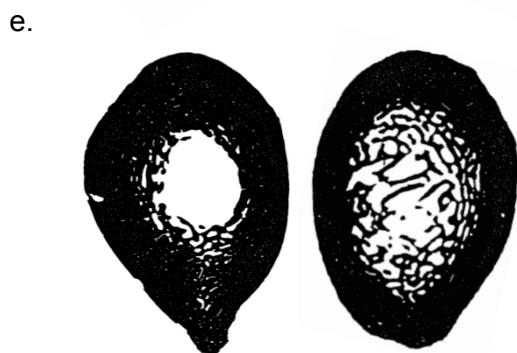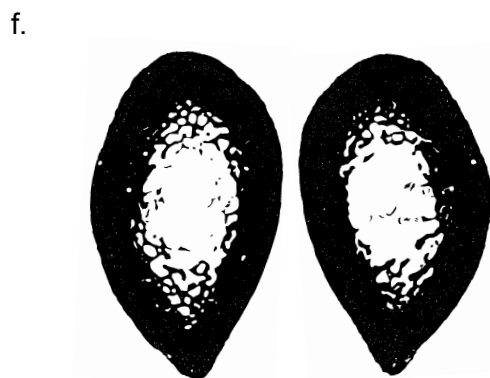

5 mm

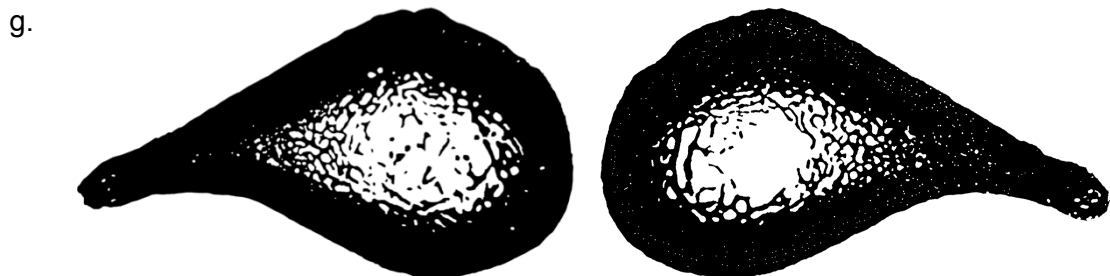

1.5 cm

Supplement: Figure S1 — 1. Binary images of thin sections produced from fossil xenarthran long bones. Catalogue numbers are provided in the respective order. (a–f) Humeri. (a–b) Glyptodon clavipes PIMUZ A/V 463 and PIMUZ A/V 465. (c) Lomaphorus ornatus PIMUZ A/V 438. (d) Pampatherium typum PIMUZ A/V 428. (e) Nothrotherium escrivanse PIMUZ A/V 477. (f) Parocnus brownii AMNH u3. (g–i) Femora. (g) Lomaphorus ornatus PIMUZ A/V 438. (h) Megalocnus rodens AMNH u2. (i) Mirandabradys zabasi AMU-CURS 128. Figure S1.2. Binary images of thin sections produced from armadillo humeri. Catalogue numbers are provided in the respective order. (a) Chaetophractus vellerosus ZMZ 20213. (b–c) Dasypus hybridus PIMUZ A/V 4798 and PIMUZ A/V 4799. (d–f) Dasypus novemcinctus PIMUZ A/V 4800, PIMUZ A/V 4801 and PIMUZ A/V 4802. (g–i) Euphractus sexcinctus ZMZ 17834, PIMUZ A/V 4803 and PIMUZ A/V 4804. (j) Tolypeutes matacus ZMZ 11151. (k–l) Tolypeutes tricinctus PIMUZ A/V 4805 and PIMUZ A/V 4806. Figure S1.3. Binary images of thin sections produced from armadillo femora. Catalogue numbers are provided in the respective order. (a) Chaetophractus vellerosus ZMZ 20213. (b–c) Dasypus hybridus PIMUZ A/V 4798 and PIMUZ A/V 4799. (d–e) Dasypus novemcinctus PIMUZ A/V 4800, PIMUZ A/V 4801. (f–h) Euphractus sexcinctus ZMZ 17834, PIMUZ A/V 4803 and PIMUZ A/V 4804. (i) Tolypeutes matacus ZMZ 11151. (j–k) Tolypeutes tricinctus PIMUZ A/V 4805 and PIMUZ A/V 4806. Figure S1.4. Binary images of thin sections produced from extant folivoran humeri. Catalogue numbers are provided in the respective order. (a) Bradypus torquatus ZMZ 11102. (b) Bradypus tridactylus NMB 10488. (c) Choloepus didactylus ZMZ 17223. (d–f) Tamandua tetradactyla NMB 10420, PIMUZ A/V 4807 and PIMUZ A/V 4808. (g) Myrmecophaga tridactyla ZMZ 11119. Figure S1.5. Binary images of thin sections produced from extant folivoran femora. Catalogue numbers are provided in the respective order. (a) Bradypus torquatus ZMZ 11102. (b) Bradypus tridactylus NMB 10488. (c) [file pone.0069275.s001.pdf]
